# The PDC30 Chatbot—Development of a Psychoeducational Resource on Dementia Caregiving Among Family Caregivers: Mixed Methods Acceptability Study

**DOI:** 10.2196/63715

**Published:** 2025-01-06

**Authors:** Sheung-Tak Cheng, Peter H F Ng

**Affiliations:** 1 Department of Health and Physical Education The Education University of Hong Kong Tai Po China (Hong Kong); 2 Department of Computing Faculty of Computer and Mathematical Sciences Hong Kong Polytechnic University Hung Hom China (Hong Kong); 3 Department of Rehabilitation Sciences Faculty of Health and Social Sciences Hong Kong Polytechnic University Hung Hom China (Hong Kong)

**Keywords:** Alzheimer, caregiving, chatbot, conversational artificial intelligence, dementia, digital health, health care technology, psychoeducational, medical innovations, language models, mobile phone

## Abstract

**Background:**

Providing ongoing support to the increasing number of caregivers as their needs change in the long-term course of dementia is a severe challenge to any health care system. Conversational artificial intelligence (AI) operating 24/7 may help to tackle this problem.

**Objective:**

This study describes the development of a generative AI chatbot—the PDC30 Chatbot—and evaluates its acceptability in a mixed methods study.

**Methods:**

The PDC30 Chatbot was developed using the GPT-4o large language model, with a personality agent to constrain its behavior to provide advice on dementia caregiving based on the Positive Dementia Caregiving in 30 Days Guidebook—a laypeople’s resource based on a validated training manual for dementia caregivers. The PDC30 Chatbot’s responses to 21 common questions were compared with those of ChatGPT and another chatbot (called Chatbot-B) as standards of reference. Chatbot-B was constructed using PDC30 Chatbot’s architecture but replaced the latter’s knowledge base with a collection of authoritative sources, including the World Health Organization’s iSupport, By Us For Us Guides, and 185 web pages or manuals by Alzheimer’s Association, National Institute on Aging, and UK Alzheimer’s Society. In the next phase, to assess the acceptability of the PDC30 Chatbot, 21 family caregivers used the PDC30 Chatbot for two weeks and provided ratings and comments on its acceptability.

**Results:**

Among the three chatbots, ChatGPT’s responses tended to be repetitive and not specific enough. PDC30 Chatbot and Chatbot-B, by virtue of their design, produced highly context-sensitive advice, with the former performing slightly better when the questions conveyed significant psychological distress on the part of the caregiver. In the acceptability study, caregivers found the PDC30 Chatbot highly user-friendly, and its responses quite helpful and easy to understand. They were rather satisfied with it and would strongly recommend it to other caregivers. During the 2-week trial period, the majority used the chatbot more than once per day. Thematic analysis of their written feedback revealed three major themes: helpfulness, accessibility, and improved attitude toward AI.

**Conclusions:**

The PDC30 Chatbot provides quality responses to caregiver questions, which are well-received by caregivers. Conversational AI is a viable approach to improve the support of caregivers.

## Introduction

### Background

How to leverage technological innovation to improve support in the protracted journey of caring for someone with dementia has been identified as one of the top priorities by dementia care researchers, practitioners, and policy makers [1,2]. Dementia family caregiving is widely regarded as a prototype of chronic stress [3,4]. With the increasing loss of abilities in persons with dementia, family members transition from a “companion” to a more heavy-duty caregiver. Initially, family members may want to know more about the condition and what to plan ahead of time. As the relative’s condition worsens, tips about handling different behaviors or situations and helping the relative with activities of daily living become more relevant, with end-of-life issues on the horizon further down the road. Thus, caregivers’ needs change over time but are often given a time-limited (eg, 8 weekly sessions) standardized training program disregarding their specific concerns [5,6]. While some open-minded caregivers may be curious to find out more about the subject matter, others feel compelled to go through materials irrelevant to their immediate concerns (or things they already know) [7]. Moreover, traditional interventions are packaged to be delivered in regular blocks of time, which may not fit into the busy and often unpredictable schedule of many caregivers [5,6]. In addition, traditional intervention modalities often rely on face-to-face contact, a problem brutally exposed during the COVID-19 pandemic [8,9], but a problem that is generally applicable to those in rural areas.

Although over two-thirds of caregivers in a large UK survey [10] indicated an interest in using technological tools to support their health and well-being, as well as their caregiving role, few such tools are available for dementia family caregivers. A major reason cited by the respondents in support of technological support was “being able to use digital tools quickly to find answers on a regular basis” [10]. Relatedly, this study explores how caregivers react to a chatbot based on generative artificial intelligence (AI) technology, which provides information and advice on demand and can be accessed anytime anywhere.

### AI Applications for Dementia Care

AI (especially natural language processing) refers to a collection of algorithms that gets computers or machines to perform autonomous actions that require human intelligence (eg, understanding complex semantics and intent, reasoning, decision-making, and self-correction). In the field of health care, the advent of AI has led to a wide range of applications, such as behavioral monitoring, risk prediction, screening, triage, diagnosis, rehabilitation, health advice chatbot, robotics, and program planning [11-21]. Such applications may be used to assist or replace human activity, or even to improve performance by enhancing efficiency and reducing human error [22-26]. Thus, AI has the potential to fill some service gaps for families with dementia as societies grapple with this public health crisis.

That said, AI is not without drawbacks. For end users, the greatest concerns are the accuracy of the output and the quality of decision-making [27,28]. An AI program is only as good as the algorithm and the data used to train it. For example, a health risk prediction program in the United States classified Black people as having lower risk profiles, compared with White people with comparable health status, resulting in many Black people being denied the proper service. The reason was due to the algorithm using health care cost as the proxy for how sick the person was, without taking into account the fact that Black people have historically been an underserved population [29].

Two reviews provide valuable insights into the development and use of AI chatbots and technology-driven solutions to support dementia care. Hoel et al [30] explored various technology-driven solutions such as social robots and tablet applications that provide activities or interactions to engage persons with dementia, most of whom live in residential care settings. Although the primary targets were persons with dementia, interactions with family caregivers were found to be enhanced through the involvement of the caregiver in the activity (eg, reminiscence therapy). Through more enjoyable interaction, the level of stress felt by family caregivers might be reduced.

The systematic review by Ruggiano et al [9] focused on chatbots, conversational AI agents that use natural language processing and large language models (both machine learning algorithms) to simulate complex human conversation [31,32]. They found 6 commercially available chatbots designed to assist people with dementia and their caregivers. Focusing on the functions and quality of these tools, their review highlighted the potential of chatbots to offer educational content for caregivers (and memory aids for patients) through accessible platforms like mobile apps and voice-activated devices. These chatbots aimed to provide users with timely information and interactive features to engage people with dementia, but a common drawback was the lack of peer-reviewed, evidence-based educational material, which undermines their trustworthiness. As a matter of fact, none of the chatbots were deployed following empirical user evaluation [9]. The information provided was often not rooted in rigorous scientific research, leaving caregivers unsure about the accuracy and reliability of the advice they receive. Based on limited programmed content, these chatbots inevitably constrain interaction with users and provide advice on a narrow range of questions [9].

Another issue is accessibility. Many chatbots require specific phrases or commands to operate effectively, limiting their flexibility and usability for a diverse range of caregivers. This rigidity can be frustrating, especially for those who are not technologically proficient. Additionally, most specialized chatbots provide only standard answers to preset questions and cannot analyze user intent and emotion. They rely heavily on users to provide specific contextual information for continued engagement.

### This Study

In view of the limitations of existing chatbots, this study reports on a newly built chatbot, called PDC30 Chatbot, which serves as a care adviser on dementia caregiving using generative AI technology and an evidence-based, comprehensive knowledge base. By simply providing information and advice, without involvement in decision-making, it avoids potential risks of AI applications mentioned above. At the same time, the use of a validated knowledge base enhances the quality of the answers and trust among users, with its information extracted by generative AI to produce a new and original response to each caregiver enquiry. As such, this new chatbot offers greater flexibility in input handling, allowing caregivers to engage in natural, conversational interactions without the need for rigid commands. Furthermore, through remarkably improved emotional and cognitive support by virtue of generative AI technology, the chatbot is capable of delivering empathetic responses and practical caregiving strategies without requiring constant caregiver input. This makes it a more autonomous and complete tool for addressing the emotional, cognitive, and practical demands of dementia caregiving.

In the following, we describe in detail the construction of the PDC30 Chatbot and its acceptability from a user perspective. A new chatbot would have little value if there is no evident advantage over other resources. Thus, the chatbot’s performance in relation to handling common questions concerning dementia and caregiving was examined by comparing its responses to those of two other chatbots including the popular ChatGPT. Upon establishing its performance value, the PDC30 Chatbot was subject to an assessment of acceptability after a 2-week use by dementia family caregivers.

## Methods

### Chatbot Development

#### PDC30 Chatbot

We use the latest GPT-4o large language model by OpenAI [33], with the following prompt to define the personality traits of the chatbot (ie, a personality agent in large language models): You are a professional counselor providing advice on caregivers’ emotional issues and caregiving challenges. The knowledge base is the Positive Dementia Caregiving in 30 Days Guidebook, an abridged and updated version of the Benefit-Finding Intervention manual (a psychoeducation program with an emphasis on searching for positive meanings in caregiving) which has been found to reduce caregiver burden and depression, with moderate to large effect sizes, up to 12 months [34-39] (the Benefit-Finding Intervention is a workshop-based program with a lengthy instruction manual written for the trainers. The manual was rewritten in simple language and a more concise form for general public consumption and became the knowledge base for this chatbot). After providing an answer, the chatbot is programmed to reference the source of the ideas, so as to encourage users to do more in-depth reading.

In other words, the chatbot would not answer any question unrelated to dementia and caregiving. As a generative AI chatbot, it can formulate answers to a wide range of questions based on these knowledge bases, rather than simply providing preset answers to selected questions. When irrelevant questions are asked, it would say “Sorry, I cannot answer your question.” The chatbot, accessible on any device with internet access (including phone, tablet, or computer), was built on botpress.com, an open-source platform for conversational AI solutions. The chatbot serves as one of the components of a self-guided, automated web-based intervention program called Positive Dementia Caregiving in 30 Days (PDC30), which is undergoing evaluation in a global randomized controlled trial (ClinicalTrials.gov identifier NCT06409455). Hence, we call it the PDC30 Chatbot. Currently, this web-based intervention program, the chatbot included, works only in the English language.

#### ChatGPT and Chatbot-B as Comparators

To evaluate how well the PDC30 Chatbot works, the same questions (see below) were fed into ChatGPT-4o (as a standard of reference) [33] and another self-constructed chatbot (called Chatbot-B for convenience). As ChatGPT is well-known, we focus on introducing Chatbot-B.

Chatbot-B’s design is identical to that of the PDC30 Chatbot, but the reference materials are different. The knowledge base for Chatbot-B consists of (1) 154 caregiving-related web pages by the Alzheimer’s Association, (2) 17 dementia caregiving-related web pages by the US National Institute on Aging, (3) 14 caregiving topic-based manuals by the Alzheimer’s Society, United Kingdom, (4) World Health Organization’s iSupport manual [40], and (5) By Us For Us Guides, a collection of 15 Canada-based documents written by people with dementia and their caregivers [41] (if a document was not optimized for automated processing, its content was manually extracted and saved as a plain text file to improve the chatbot’s ability to interpret and analyze the information). In other words, Chatbot-B represents a rather comprehensive and authoritative knowledge base from which answers are drawn. In this sense, Chatbot-B is like an assistant surfing the internet for relevant materials on behalf of the user and summarizes the main points for the user in far less time than the user surfing the internet himself or herself.

Together, ChatGPT and Chatbot-B provide strong reference points for assessing the quality of PDC30 responses. ChatGPT is an existing tool that can be used by caregivers in countries where it is available. It is trained on an enormous text retrieved from the internet but is known to have the risk of providing false information when such information exists on the internet. This reliance on internet-based sources makes it vulnerable to inaccuracies, particularly when it draws from unreliable content. Additionally, the chatbot may struggle with context or nuance, offering responses that are overly general or missing critical domain-specific details, which can be problematic in sensitive caregiving scenarios [9]. Chatbot-B, on the other hand, is a new chatbot dedicated to dementia and caregiving topics using authoritative materials. A caveat needs to be mentioned. There are pros and cons of including many reference materials. Up to a certain point, the benefit of including more texts levels off given the redundancy across their content. Processing time may be lengthened as the knowledge base expands in size. Different texts may also offer advice that contradicts each other. Hence, a chatbot based on a single text (ie, the PDC30 Guidebook) may not necessarily fare worse.

#### Testing Materials and Chatbot Responses

We constructed 21 questions commonly asked by family members by surfing the internet and subjected the three chatbots to the same questions to see how their answers compare. The responses provided by PDC30 Chatbot and Chatbot-B are reproduced in Multimedia Appendix 1, whereas those by ChatGPT are shown in Multimedia Appendix 2. Note that due to the use of generative AI, the answers provided to the same question will vary slightly from time to time; thus, these are not fixed answers, but rather one sample of a range of possible answers by the respective chatbot. Moreover, it should be mentioned that the boldfaced headings in front of the bullet points were created by the chatbots; they did not exist in the original sources. Interestingly, at one point, the PDC30 Chatbot addressed the first author (“Dear Tak,” response to the question “Why is my wife acting like a different person”) and that was the only instance any of the chatbots addressed the user by name when answering questions.

For PDC30 Chatbot and Chatbot-B, one can see that despite using proper nouns (eg, mom and husband) and pronouns (eg, her), the chatbots had no problem understanding they were the care recipients. The wording in some questions was intentionally nonspecific (to mimic everyday conversation) and GPT-4o, when constrained to answer questions related to dementia and caregiving, had no difficulty grasping the meaning in the context of dementia caregiving. On the contrary, ChatGPT expectedly needed information on the specific context to construct more relevant responses (for instance, when asked about preparation for the future, it talked about financial investment unless being told that the question concerned a family having a relative newly diagnosed with dementia). Hence questions for ChatGPT were elaborated accordingly. By comparison, questions posed to PDC30 Chatbot and Chatbot-B mimicked natural conversations a lot more.

In terms of answers, occasionally there are bullet points, the relevance of which to the question is not immediately apparent (eg, Chatbot-B’s points “utilize online services” and “stay engaged and active” to the question “What preparation should we make for the future?”). This issue appeared to be more common for ChatGPT, including, but not limited to: considering home safety and daily activities for the preparation for future question; encouraging activity involvement for the question on communication skills; discussing financial or legal planning and communication skills for the question on managing personal feelings and stress; asking the caregiver to join support groups, find respite, consider environmental safety, and to take breaks for himself or herself when the question was how to deal with mom’s apathy; and getting adult day care, power of attorney, and medical directives when being asked how to allow husband to wander safely. Such answers may well confuse caregivers, limiting the value of ChatGPT as a consultation resource.

Moreover, ChatGPT responses tended to be repetitive, while missing some key advice to caregivers, such as avoiding confrontation and giving due recognition to the disease as the real causal agent for problematic behaviors. On the whole, the responses by PDC30 Chatbot and Chatbot-B were more concise, specific, and to the point. Considering the fact that these chatbots were providing advice on complicated matters, we think their overall performance was quite good—the results support chatbots using generative AI technology as a viable approach to offer advice to caregivers, and the results are better with topic-focused chatbots. For this reason, we focus on the relative performance of PDC30 Chatbot and Chatbot-B below.

The answers provided by these two chatbots were surprisingly similar. In fact, the answers to the question “I am mad with myself; I made so many mistakes” were identical between the two chatbots, though PDC30’s answer was probably clearer with more elaboration. Though there is no straightforward way to ascertain the relative quality of the answers, we are of the opinion that compared with PDC30 Chatbot, Chatbot-B’s performance was inferior in relation to the questions “I cannot accept the idea that my Mom has dementia and may leave me one day” and “I am so frustrated I can’t control my emotions; I would even take out my irritation on her.” One difference between the two chatbots does stand out, which is that when it comes to questions related to emotional or mental health issues (technically the above question about not being able to accept mom’s condition is also an emotionally laden question), the PDC30 Chatbot provided a greater variety of suggestions. For example, pleasant event scheduling and alternative thinking are important coping strategies but were mentioned by the PDC30 Chatbot only. Other strategies including positive meaning-making and tackling unrealistic assumptions about the care recipient were also emphasized a lot more by the PDC30 Chatbot. These differences are notable, as psychoeducational interventions that include tactics taken from psychotherapy have been found, in a comprehensive meta-analysis of 131 randomized controlled trials, to be much more beneficial for dementia caregivers (especially in terms of relieving depression and promoting self-efficacy and positive gains) than psychoeducation without such tactics [2]. On the whole, the results support the value of the PDC30 Chatbot as a “counselor” for dementia caregivers. Typically, an answer was provided in 20 seconds or less, despite the volume to be scanned, suggesting that a generative AI chatbot is a rather convenient way for caregivers to obtain basic information and advice.

Some brief remarks about Chatbot-B are warranted before moving on to the main acceptability study. In terms of referencing, Alzheimer’s Association materials were used in formulating responses to 14 of the 21 questions; Alzheimer’s Society and By Us For Us documents were each used for 12 questions; National Institute on Aging web pages, 9 questions; and iSupport, 8 questions. Thus GPT-4o did scan the entire knowledge input before drafting its answers. Note also that Chatbot-B works only if the URLs are up to date. This can make it a less preferable choice as an intervention tool, compared with the PDC Chatbot which has a stable and well-defined knowledge source.

### Acceptability Study for PDC30 Chatbot—Design and Procedure

In light of the positive results for the PDC30 Chatbot, we proceeded to the next phase to evaluate the acceptability of the chatbot. A mixed methods study was conducted, in which caregivers were shown the PDC30 Chatbot’s responses to the 21 questions above, and asked to use the chatbot (accessible using a hyperlink) for two weeks, at least once a day (as this study was focused on the evaluation of the chatbot, the other components of the PDC30 intervention were not included). At the end of 2 weeks, participants indicated, on a self-report questionnaire, their frequency of using the chatbot on a scale of 1=almost never, 2=several times a week but less than daily, 3=once a day, and 4=more than once a day. They rated the chatbot using the following questions: (1) the chatbot is easy to use, (2) the answers are easy to understand, (3) the answers are helpful, (4) overall, you are satisfied with the chatbot, and (5) you would recommend the chatbot to other caregivers (all rated from 1=strongly disagree to 5=strongly agree). Participants were also asked to write down any other thoughts they had about the chatbot; this written feedback was subject to thematic analysis [42].

### Participants

Participants were recruited through posting notices on campus. The inclusion criteria are (1) aged 18 years or older, (2) self-identification as providing care to a relative with dementia, and (3) self-reported fluency in English. There was no exclusion criterion. All participants were relatives of students and staff who were taking care of a family member with dementia and who provided informed consent to participate. We stopped recruitment after the 21st participant because data saturation had evidently been reached, as the same themes kept repeating. Participants were not financially compensated but were given continuing access to the PDC30 Chatbot after the study.

### Ethical Considerations

The study was approved by the Human Research Ethics Committee of The Education University of Hong Kong (reference 2021-2022-0077). Written informed consent was obtained from all participants.

### Data Analysis

The two authors independently read the participants’ written feedback to first become familiar with the entire set of qualitative data. After highlighting key points and making notes, they generated initial codes for each written feedback. Themes were then extracted by identifying similar codes and patterns using an inductive approach. The two authors compared their work and found that similar themes were identified. The themes were finalized and articulated [42]. As for the Likert-type questions, data were summarized using descriptive statistics.

## Results

### Overview

Participant characteristics and their ratings of the chatbot can be found in Table 1. Understandably, participants were predominantly child caregivers (10 daughters and 4 sons). Two caregivers were the daughters-in-law of the care recipient. Only four were spouses (one wife and three husbands) and one was a sister.

As can be seen from Table 1, the responses were overwhelmingly positive. A total of 15 out of 21 (71%) participants used the chatbot more than once per day (overall mean 3.62, SD 0.67). Participants thought the chatbot was very easy to use and most gave the highest rating (mean 4.52, SD 0.68). They also thought the advice provided was quite helpful (mean 4.29, SD 0.56) and relatively easy to understand (mean 3.81, SD 0.98). Given such positive experiences, it is no wonder that they felt rather satisfied with the chatbot (mean 4.05, SD 0.59). A total of 16 out of 21 (76%) participants would recommend or strongly recommend it to other caregivers (mean 4.24, SD 0.83). These ratings were more or less substantiated by the participants’ written qualitative feedback (Table 2). Thematic analysis of the written comments revealed 3 recurring themes.

**Table 1:** Acceptability study sample and their ratings of the PDC30 Chatbot.

Caregiver	Age	Sex	Relationship	Q1^a^	Q2^b^	Q3^c^	Q4^d^	Q5^e^	Q6^f,g^
A	40	F	Child	4	5	4	5	4	5
B	58	M	Child	4	4	5	4	4	5
C	51	F	Child	4	5	5	5	5	4
D	36	F	Child	3	5	5	4	4	5
E	73	F	Spouse	2	5	2	3	3	3
F	50	F	Child	4	5	5	4	5	5
G	70	M	Spouse	4	5	3	4	5	4
H	46	M	Child	4	4	4	4	4	5
I	42	F	Child	4	5	4	5	4	5
J	50	F	Child	4	4	3	5	4	5
K	43	F	Child-in-law	4	5	5	4	3	4
L	44	F	Child	3	4	3	4	4	4
M	48	F	Child	2	5	4	5	3	3
N	55	F	Child-in-law	4	5	5	4	4	5
O	50	F	Child	3	4	4	4	5	5
P	72	M	Spouse	4	4	3	4	4	5
Q	71	M	Spouse	4	3	2	4	4	4
R	54	M	Child	4	5	4	5	4	3
S	68	F	Sibling	4	3	3	5	4	3
T	50	M	Child	4	5	4	4	4	3
U	55	F	Child	3	5	3	4	4	4
Mean (SD)	53.62 (11.18)	—^h^	—	3.62 (0.67)	4.52 (0.68)	3.81 (0.98)	4.29 (0.56)	4.05 (0.59)	4.24 (0.83)

^a^Q1: frequency of use.

^b^Q2: whether easy to use.

^c^Q3: answers easy to understand.

^d^Q4: answers helpful.

^e^Q5: satisfaction.

^f^Q6: likelihood to recommend to others.

^g^Except for Q1 which is scored 1-4, all other questions are scored 1-5, with higher scores indicating a better experience.

^h^Not applicable.

**Table 2:** Participants’ written comments (reproduced in full) about the PDC30 Chatbot.

Caregiver	Written comments
A	I love it! I have to work and take care of my mom. My sister do not help. I feel very lonely and not sure what to do at times. Now I can have this chatbot to talk to any time I want - it feels like a friend. And it gives me ideas about how to handle my mom too.
B	Don’t feel I should go get help from the centre’s social worker again. The chatbot’s advise is better! And it works 24 hours a day! I actually feel so stupid now that I never trusted AI. I am really surprised how good it is.
C	The answer refer to a guidebook but we don’t have it. That part is a bit confusing. That said, I really like the fact that I can now ask anything any time.
D	Initially I have reservation. I have tried many chatbots (like banks) and they are so annoying because they pretended to help but only gave you standard replies not addressing your query. This one is different. Impressed!
E	I am very busy because my husband fell. So I don’t use it much. I appreciate the advise but some suggestions are not easy to follow. But I find it very easy to use. My first thought it is strange talking to a robot. But after using it, I don’t feel that anymore. When ok, I will use it more.
F	Can I keep using the chatbot, please? When my nephew mentioned this chatbot to me, I thought it is a joke. Talking to a machine? But I use it every day now. Please let me use it.
G	How come not available earlier? I wish had it 2 years ago when I was most helpless!
H	Did Tom Cruise’s boss said drones do not sleep but pilots do? Well, this is a very good drone.
I	This is the first time a chatbot is REALLY answering my questions! There is some wait but waiting time reasonable.
J	Professor, thanks indeed for giving me a chance to use this chatbot. It is very helpful!
K	Most answers are relevant and informative. Quick too. Better than I thought.
L	It say it is not a therapist but it is a therapist. It help me calm down when I was very very frustrated! Thanks. I will try to use it more.
M	A good companion. Answer quality is good. But I use it only when I could not find someone to talk to. It’s a backup when no one is available.
N	I can screen cap the answer and send to my husband. Then we discuss how to handle my mother-in-law. I feel more confident because I have both knowledgeable advice and husband support.
O	Frankly, I was very skeptical in the beginning. But I gradually like it more. I have a smart speaker but it does not give good answer to this type of questions.
P	Never used a chatbot. Very interesting. Good information. My wife doesn’t know what I do on my phone when I am looking for help. If I call my daughter, she would hear what we said and feel upset.
Q	Some terms are difficult. I need to check dictionary. But very convenient. 24 hour service. My wife has frequent difficult behavior and I like the suggestion to ignore or take a break. Some tips for bathing and serving meal helped.
R	It’s a good chatbot. I love it but 2 weeks too short to say how good it really is.
S	Not used to talk to machine. Often I have a problem but forget the chatbot is there. Forget can use my phone. Then afterwards when I see my phone, I remember! Then I ask question and get some helpful answer but sometime too late.
T	I work in IT field and so this is no surprise to me. I am glad something like this is finally available because caregiving is hard! I did not get any nonresponse and response time is very good. The answers are specific with enough elaboration and minimal repetition. These are what determine good user experience.
U	Amazing. It answer all my questions so far. But my father is mild stage. May be my questions not hard enough. Interesting to see if it can help me when things get more tough.

### Theme 1: Helpfulness

This is the most dominant theme which was explicitly (caregivers A, B, I, J, L, K, M, N, P, Q, and U) or implicitly (caregivers D, F, G, H, O, R, S, and T) mentioned by 19 (90%) participants. Four (caregivers D, I, O, and T) said the chatbot’s answers were appropriate, informative, and specific, unlike other chatbots that provided inadequate, irrelevant, or standardized replies. Caregiver L specifically mentioned that the chatbot helped her to calm down when severely frustrated. Her comments were echoed by caregiver Q who also referred to helpful practical suggestions by the chatbot. Caregiver F, despite being informed at the study’s outset, forgot that the chatbot would continue to be available, and asked if she could keep on using it after the testing period, a gesture suggestive of the helpfulness of the chatbot. Another participant (caregiver G), who cared for his wife, lamented the late coming of this resource and imagined what difference it would have made to his situation if it had been available 2 years earlier, again testifying to the perceived helpfulness of the chatbot. By comparing the chatbot to a “very good drone,” caregiver H might be saying that the chatbot got the job done (ie, providing good advice), a point verified in a subsequent email conversation with the caregiver. Perhaps a bit exaggerating, caregiver B even complimented the chatbot by saying that its advice was better than the social worker’s. One wife caregiver (E), however, thought that some of the suggestions by the chatbot were not easy to follow, which was not entirely surprising as behavioral and cognitive change takes time and participants did not have the full intervention program to assist them. On the positive side, this comment suggests that the answers were relevant, just not easy to follow through.

### Theme 2: Accessibility

In total, 11 (52%) caregivers mentioned, in one way or another, the usability of the chatbot, including being user-friendly (caregiver E), lack of (or low rates of) nonresponse (caregiver T), reasonable waiting time before getting a response (caregivers I, K, and T), and most of all, the chatbot’s 24/7 accessibility (caregivers A, B, C, H, and Q). Caregiver H even paraphrased a conversation in the movie “Top Gun: Maverick” to illustrate this point.

Unexpectedly, two caregivers mentioned functional merits unrelated to the chatbot itself. Caregiver N talked about the convenience of saving the chatbot’s responses and sharing them with other family members. This action, as alluded to by this caregiver, might encourage more involvement by other family members and support to the main caregiver. In addition, caregiver P made an interesting point about the privacy afforded by using the chatbot on his smartphone. His comment was a reminder of the dilemma faced by many caregivers, especially those in crowded living conditions such as the case in Hong Kong, when trying to seek help when the care recipient is nearby. Conversations, whether over the phone or face-to-face, may be overheard by the care recipient, who, in turn, reacts with more behavior problems. The chatbot operated on the phone offers caregivers private space to get help when necessary.

### Theme 3: Attitude Toward AI

Five participants (caregivers B, D, E, F, and O) expressed that they were initially skeptical of AI but the experience changed their perception of it. For example, caregiver B started with a mistrust of AI while caregiver F initially found seeking advice from a machine a ridiculous idea. After trying it, both became excited by the technology and were using the chatbot more than once a day. A related sentiment was expressed by another caregiver, S. She was not used to talking to a machine, and after having access to the chatbot, kept forgetting about it. Yet, the undertone of the feedback was that she found the chatbot responses to be useful and lamented that occasionally she had not taken timely advantage of the resource.

Not everyone was as receptive to the technology. Caregiver M did not reject the chatbot but reserved it for occasions when she could not find people to talk to. Nevertheless, the feedback overall suggested that experience with the chatbot induced a favorable attitude toward it in those who questioned its value to begin with.

## Discussion

### Principal Findings

This study demonstrates that using a chatbot to help dementia caregivers is a viable approach. Taking advantage of AI development, we were able to build the PDC30 Chatbot that functions as a “counselor” to caregivers (ie, its purpose), by applying a personality agent to constrain the chatbot’s behavior and by feeding it with an appropriate knowledge base. The generative AI algorithm summarized the points using “its own” words and organized them using headings to facilitate reading. At first, it might seem that feeding it with more resource materials would help it formulate better responses—for this reason, two other chatbots were included for comparison, namely ChatGPT and Chatbot-B incorporating insider perspectives (those from patients and caregivers) and guidelines by several authoritative agencies. In our testing using 21 common questions by caregivers, there was indeed a tendency for Chatbot-B to generate slightly longer responses than PDC30 Chatbot, though this was not always the case. Nevertheless, length per se does not determine quality, as longer answers may contain irrelevant and repetitive points, as we have seen, especially in the case of ChatGPT. On the whole, we think that PDC30 Chatbot and Chatbot-B were superior to ChatGPT and performed similarly, with PDC30 having a further edge on emotional and mental health issues. In addition, the PDC30 Chatbot was favorably received in an acceptability study of 21 dementia family caregivers after using it more than once a day for two weeks. The chatbot was considered user-friendly, with its responses helpful and easy to understand. Their written feedback about the chatbot centered on three themes: it was helpful and accessible, and it improved their perception of AI as a helping agent. Overall, the experience was rather satisfactory and the participants would strongly recommend it to other caregivers.

Both PDC30 Chatbot and Chatbot-B provide citations of the sources they refer to when formulating responses. A caveat needs to be mentioned. While the citation system may prompt caregivers to do more reading of the relevant materials, it is good only to the extent that the sources can be readily located. As explained in Table 1 footnote, GPT-4o cannot name the author of the web pages cited. If the source is a document, this was handled by the way the document is named, such as “Alzheimer’s Society Making Your Home Dementia Friendly”—in other words, the citation is to the name of the file used. If it is just a web page, GPT-4o provides a citation using the first title that appears on the web page; the agency or author producing the web page is omitted—this is primarily due to limitations in GPT-4o’s design and functionality. First, GPT-4o cannot read logos or metadata from images on web pages, where authors’ names and relevant citation details are often embedded. If the website does not contain alternative text, GPT-4o cannot read the images. Second, while the model processes textual data, it may only effectively distinguish between content and metadata, such as author names or publication details, if explicitly mentioned in the text. The website needs to state the metadata clearly. Additionally, the training data (ie, web page metadata) may not include structured citation formats, making it difficult for the model to recognize and extract authors’ names reliably. Furthermore, the design of GPT-4o may include privacy considerations that prevent it from naming individuals unless explicitly included in the training data or provided in a clear, structured format. To address this issue, converting all relevant web pages into text files with clear filenames and structured citation information can help ensure that author names and other citation details are correctly identified and referenced by the model. On the contrary, the PDC30 Chatbot is to be used together with the Guidebook in actual operation—both are to be hosted on the same intervention website. Thus, the citation problem mentioned above does not apply.

Perhaps a more important consideration in terms of operation is that Chatbot-B requires constant monitoring and updating, should organizations add new web pages, remove certain web pages, or change the URLs of the web pages—there are indeed a very large number of web pages to monitor. Worse, changes to the content of a web page with the same URL will be hard to detect, unless a “last updated date” is provided. By comparison, the PDC30 Chatbot is much easier to maintain, as it is based on only one text.

The PDC30 Chatbot was built with the intention to be included in a randomized controlled trial, and so we subject it to an evaluation of acceptability by caregivers. All but one caregiver provided highly positive feedback about the chatbot; the only exception came from a wife caregiver whose use of the chatbot was disrupted by her husband’s fall early in the trial period (had she contacted the research team then, we would have adjusted her trial period so that she would have been able to try the chatbot at a more convenient time. Unfortunately, we were not aware of the issue until she turned in her responses). On the whole, the caregivers found the chatbot easy to use (including the wife caregiver mentioned above) and the answers were informative, relevant to their needs, and easy to understand. Many particularly appreciated the chatbot’s helpfulness and 24/7 accessibility, with some further commenting on its lack of nonresponse and reasonable waiting times before responses were generated. As a result, some caregivers acquired a much more favorable attitude toward AI technology because of using the chatbot. The great majority reported high levels of satisfaction with the chatbot and would recommend it to other caregivers. Thus, the PDC30 Chatbot was well-received by caregivers who used it. The chatbot’s frequent use was also a testimony to its reception among the caregivers.

### Limitations

Despite the encouraging findings, a few limitations need to be mentioned. First, the sample was predominantly adult children. We do not think the paucity of spousal or sibling caregivers was due to the relative lack of digital literacy among older caregivers as there is no skill required to use the chatbot (other than typing questions into the input space). The older, spousal or sibling caregivers who participated generally found the chatbot easy to use. Rather, we think that this reflects cohort differences in the preference for digital material [43]. Furthermore, the composition of the sample is not as one-sided as it seems, as about two-thirds of the caregivers in this community are children [44]. That said, there is no doubt a certain degree of self-selection as not everyone prefers digital resources (we did have two caregivers in the sample who were initially doubtful of the technology). A future study focusing on older caregivers, especially those with little digital literacy, is warranted.

Second, the chatbot was constructed to speak English only, in alignment with the language used in the parent intervention program being evaluated in a global trial. It is probably too harsh to consider this a limitation but in the context of this study, fluent English-speaking persons do not represent the caregiver population in Hong Kong. Due to the time pressure to launch the clinical trial, we also did not recruit caregivers from English-speaking countries. Although the generalizability of the findings may be limited, the PDC30 Chatbot’s responses shown in Table 1 suggest that the chatbot should be quite suitable for caregivers in other countries as well.

### Conclusions

To the best of our knowledge, this is the first study reporting the development of a psychoeducational chatbot for dementia caregivers and testing its functioning and acceptability among caregivers. The convenience of delivering advice to caregivers by AI-driven chatbots is an approach that needs to be explored further by the field. Services for families with dementia are lacking, especially in resource-poor countries, and even when services are available, many caregivers do not use the services due to lack of time, services not meeting their needs, or simply wanting to locate information themselves [45]. Good chatbots may well fill some of the gaps and get help to these caregivers around the world. There is one more important feature that makes chatbots an advantageous option—only small amounts of data are transmitted each time and hence chatbots are especially suitable for low-income countries and rural areas in general where internet coverage remains an issue.

## Appendix

Multimedia Appendix 1 Responses provided by PDC30 (Positive Dementia Caregiving in 30 Days) Chatbot and Chatbot-B.

Multimedia Appendix 2 Responses provided by ChatGPT.

## Abbreviations

AI: artificial intelligence
PDC30: Positive Dementia Caregiving in 30 Days

## Data Availability

All data generated or analyzed during this study are included in this published article.
