# Supplementary material for: The PDC30 Chatbot—Development of a Psychoeducational Resource on Dementia Caregiving Among Family Caregivers: Mixed Methods Acceptability Study
Source: JMIR Aging. 2025 Jan 6;8:e63715. doi: 10.2196/63715 (PMC11758934; doi:10.2196/63715)
Supplement: Multimedia Appendix 1 [file aging_v8i1e63715_app1.docx]

| Question/conversation | PDC30 Chatbot | Chatbot-B |
| --- | --- | --- |
| What is dementia? | Dementia is a condition that affects various mental capacities, such as memory, attention, navigation, reasoning, judgment, perceptual organization, motor planning, and the ability to recognize thoughts and emotions in others. It can have a significant impact on people's memory, their ability to focus on complex tasks, express themselves, understand messages, recognize faces and objects, perform familiar actions, be aware of how others perceive their behaviors, and make decisions. Dementia is a broad term that encompasses a range of diseases that cause damage to brain cells, resulting in these impairments.  Source: (document) PDC30 Guidebook Module 1.3.docx , (document) PDC30 Guidebook Module 1.1.docx | Dementia is a term used to describe a decline in mental ability that is severe enough to interfere with daily life. It includes the loss of memory, language, problem-solving, and other thinking skills. Dementia is not a normal part of aging and is caused by damage to brain cells, which affects their ability to communicate. This can impact thinking, behavior, and emotions. Alzheimer's disease is the most common cause of dementia, accounting for 60-80% of cases.  Source: (webpage) Veterans and Dementia , (webpage) 10 Healthy Habits for Your Brain , (webpage) What Is Dementia? Symptoms, Types, and Diagnosis , (webpage) What Is Dementia? , (webpage) Dementia vs. Alzheimer’s Disease: What is the Difference? , (webpage) What is Alzheimer’s? |
| I cannot accept the idea that my Mom has dementia and may leave me one day. | Coping with the idea that your mom has dementia and may leave you one day can be quite challenging. Here are some strategies that might be helpful:   - **Mindset Adjustment**: Adjust your expectations and beliefs to reduce stress and improve your emotional well-being. - **Understanding the Disease**: Recognize that the changes in your mom's behavior are a result of the disease affecting her brain. This understanding can help you manage your expectations and responses. - **Positive Aspects of Caregiving**: Many caregivers have found positive aspects in their role, which can bring meaning and satisfaction. - **Seeking Support**: Reach out to family, friends, or neighbors for temporary caregiving assistance, allowing you to take a break. - **Emotional Control**: Practice tolerance and patience. Staying calm is important, as anger can harm your heart and hurt your loved one. - **Letting Go**: Accepting that a part of your relative is gone is a long and painful process, but it is essential for positive adjustment. - **Finding Solace in Small Gestures**: Simple gestures like touching and looking into your mom's eyes can remind you of the deep connection between you.   These steps can help you manage your emotions and stress more effectively while taking care of yourself.  Source: (document) PDC30 Guidebook Module 1.4.docx , (document) PDC30 Guidebook Module 1.1.docx , (document) PDC30 Guidebook Module 1.5.docx | It can be really tough to come to terms with the fact that someone you love who has dementia may eventually leave you. The diagnosis of dementia brings a sense of anticipated loss and grief, which can trigger feelings of guilt, confusion, depression, and anger if not acknowledged and validated. Dementia is a progressive condition, meaning that the person will gradually lose their memory, independence, and personality. This can be incredibly overwhelming and make you feel like you're losing the person you once knew. On top of that, the uncertainty of the future and the changes that come with the disease can be daunting, making it difficult to cope with the idea of long-term care and the potential need for more support.  Source: (document) By Us For Us_Loss & grief.txt , (document) Alzheimer's Society_Carers look after yourself.txt |
| How do I know what to expect, and how fast will things change? | The progression of dementia varies greatly among individuals, and it is not possible to predict with certainty how fast things will change. Most dementias, including Alzheimer's disease, worsen gradually over time, while others, like vascular dementia, may have a more abrupt onset and change with each incident of stroke.  The symptoms and their severity can fluctuate, with moments of clarity interspersed with periods of confusion. Caregivers need to be flexible and adapt activities to the changing abilities of the person with dementia.  As the disease progresses, the caregiving role will become more demanding, requiring close supervision and assistance with everyday tasks. It is important to set realistic expectations and focus on improving the quality of life for your loved one.  Overall, the journey of dementia caregiving is marked by continuous adaptation and problem-solving.  Source: (document) PDC30 Guidebook Module 1.5.docx , (document) PDC30 Guidebook Module 1.2.docx , (document) PDC30 Guidebook Module 2.3.docx , (document) PDC30 Guidebook Module 1.1.docx , (document) PDC30 Guidebook Module 2.2.docx | Dementia is a progressive condition, which means that most symptoms will worsen over time. The rate of progression can vary greatly from person to person. Some individuals may require significant support soon after diagnosis, while others may maintain a level of independence for many years.  As dementia progresses, the person will require more assistance with daily activities and personal care. Predicting the progression can be challenging as it depends on factors such as how the disease spreads in the brain, as well as the individual's personality, life experience, and support systems.  Recognizing that dementia affects each person differently and that the progression can occur gradually or in stages can aid in planning and adapting to the changes.  Source: (document) Alzheimer's Society_Dementia guide.txt , (document) Alzheimer's Society_Caring for person with dementia practical guide.txt |
| What preparation should we make for the future? | To prepare for the future, consider the following steps:   1. **Early Diagnosis**: Getting an early diagnosis of dementia allows more time to prepare for caregiving responsibilities and helps avoid unrealistic expectations and blame. 2. **Asset Management and End-of-Life Decisions**: Make decisions about managing assets and end-of-life care while the person still has the mental capacity. This includes assigning someone to handle finances and making advance directives, such as whether to resuscitate in case of cardiac or respiratory arrest. These decisions should be legally or formally registered. 3. **Power of Attorney**: Establish enduring or lasting power of attorney to handle future decisions. The laws for this vary by country, so make sure to check the specific regulations in your area. 4. **Avoid Big Promises**: It's important to avoid making big promises, such as never sending your relative to an institution, as circumstances may change. Instead, have open discussions about different scenarios and their pros and cons while your relative is still capable.   Following these steps will help ensure that both you as the caregiver and the person with dementia are better prepared for the future.  Source: (document) PDC30 Guidebook Module 1.1.docx | - **Plan ahead as soon as possible**: It's crucial to plan ahead and have conversations with your loved ones about the future as early as you can. - **Take care of financial and legal matters**: Make sure your finances and legal affairs are in order. This includes creating a will, setting up a Lasting Power of Attorney, and organizing important documents like bank accounts, tax information, benefits, pension details, mortgage or rent documents, insurance policies, and your will. - **Consider advance care planning**: Discuss and document your preferences for future care, including advance statements and advance decisions to refuse treatment. - **Seek professional advice**: Consult with a lawyer, banker, or financial advisor to get answers to your questions about planning for the future. - **Organize and review important documents**: Ensure that all your paperwork is in one place or a secure location known to those you trust. - **Explore long-term care options**: Prepare for inevitable changes and consider options such as home care or moving to a long-term care facility. - **Discuss your future care preferences**: Have conversations with your family and healthcare or social care professionals about your preferences for future care. - **Utilize online services**: Consider using online banking and other digital services to make managing your finances easier. - **Set realistic goals**: Establish daily and future goals, and reward yourself for achieving them. - **Stay engaged and active**: Find ways to stay involved and active, such as joining a support group or participating in activities.   These steps can help you navigate the situation more effectively while maintaining your dignity and comfort.  Source: (document) Alzheimer's Society_Dementia guide.txt , (document) By Us For Us_Loss & grief.txt , (document) By Us For Us_Before_early diagnosis.txt , (document) Alzheimer's Society_Caring for person with dementia practical guide.txt , (document) Alzheimer's Society_Understanding & supporting person with dementia.txt |
| Why is my wife acting like a different person? | Dear Tak, it's not easy to see your wife acting like a different person. Dementia can bring significant changes in personality and behavior. These changes occur because the disease affects the brain, causing symptoms like memory loss, confusion, and altered perceptions of reality. For instance, she may not recognize familiar faces, including her own, and may display agitation, irritability, or even aggression at times.  Moreover, dementia can lead to disinhibition, causing her to say or do things that are socially inappropriate or out of character. It's important to remember that these behaviors are not intentional but a result of the disease's impact on her brain. Understanding this can help you manage your expectations and respond with empathy and patience.  Source: (document) PDC30 Guidebook Module 1.3.docx , (document) PDC30 Guidebook Module 1.1.docx , (document) PDC30 Guidebook Module 1.2.docx | When someone has dementia, it's not uncommon for their personality and behavior to change. This is because the disease affects the brain, causing symptoms like memory loss, confusion, and altered perceptions of reality. It's important to understand that these changes are a result of the disease, and not something your wife can control. By recognizing this, you can better manage your expectations and respond to her in a supportive way.  Source: (document) By Us For Us_Loss & grief.txt , (document) Alzheimer's Society_Understanding & supporting person with dementia.txt |
| How can I communicate with my wife? | - **Keep it Simple**: Use simple language and clear instructions to avoid confusion. - **Break Tasks Down**: Divide activities into smaller steps and guide her through each one. - **Provide Cues**: Use verbal and nonverbal cues to help her understand and follow instructions. - **Be Patient**: Give her extra time to process instructions and respond, and avoid rushing her. - **Engage in Joint Activities**: Participate in activities she enjoys and can still do, like looking at photos or doing simple chores. - **Use Touch**: Physical touch, like holding hands or hugging, can be a powerful way to connect when verbal communication is difficult. - **Maintain a Routine**: Establish a regular schedule to provide structure and reduce confusion. - **Avoid Arguments**: Instead of arguing, try to understand her perspective and provide reassurance. - **Use Music and Photos**: Engage her with her favorite music and go through photo albums to trigger memories and create a sense of connection.   These strategies can help improve communication and maintain a meaningful connection with your wife.  Source: (document) PDC30 Guidebook Module 2.2.docx , (document) PDC30 Guidebook Module 1.5.docx , (document) PDC30 Guidebook Module 2.3.docx , (document) PDC30 Guidebook Module 2.4.docx | - Keep your sentences simple and easy to understand. - Make sure to maintain eye contact and position yourself at eye level. - Avoid sudden movements and tense facial expressions, as they can be overwhelming. - Find a comfortable spot where she can see and hear you clearly. - Show that you are actively listening by giving her your full attention. - Try to ask one question at a time, using simple yes or no options. - Use gestures, facial expressions, and body language to enhance communication. - Minimize distractions by turning off the TV or radio. - Be patient and give her plenty of time to respond without interrupting. - Use gentle touch to get her attention and create a sense of connection. - Feel free to share these tips with others who interact with her.   Source: (document) Word Health Organization iSupport manual.txt , (document) Alzheimer's Society_Understanding & supporting person with dementia.txt , (document) Alzheimer's Society_Your relationships booklet.txt , (document) By Us For Us_Enhancing communication.txt , (webpage) Communication and Alzheimer’s |
| How can I manage my own feelings and stress? | To take care of your own emotional well-being and manage stress, here are some strategies you can consider:   1. **Pleasant event scheduling**: Make sure to plan and engage in enjoyable activities at least once a day. 2. **Relaxation**: Practice relaxation techniques like slow breathing regularly. 3. **Intention and motives**: Reflect on your reasons for being a caregiver and reconnect with them. 4. **Support seeking**: Don't hesitate to ask for help and support; you don't have to do it all alone. 5. **Mindset adjustment**: Challenge and modify any negative assumptions, beliefs, or attitudes you may have, including unrealistic expectations of the person you're caring for.   In addition, you can:   - **Practice self-distraction**: Shift your focus away from stressful situations and redirect it towards something more pleasant, like a hobby or a simple task. - **Increase patience and tolerance**: It's important to maintain emotional control even when faced with challenging situations. - **Use humor and find meaning**: Try to find humor in difficult situations and reframe them in a positive light. - **Take breaks**: If you're feeling overwhelmed or annoyed, take a break or shift your attention to something else to help calm yourself down. - **Seek social support**: Reach out to someone you trust and talk about your feelings when you're feeling helpless or stressed. - **Engage in alternative thinking**: Look at situations from different perspectives to find alternative ways to respond.   By following these steps, you can effectively manage your emotions and stress while taking care of yourself.  Source: (document) PDC30 Guidebook Module 1.3.docx , (document) PDC30 Guidebook Module 1.5.docx , (document) PDC30 Guidebook Module 1.4.docx | To take care of your emotional well-being and cope with the challenges of caregiving, here are some strategies you can consider:   1. **Identify sources of stress**: Take note of what triggers your stress and try to avoid or minimize these situations whenever possible. 2. **Address triggers**: Work on changing your perspective towards stressful situations and try to find positive aspects in them. 3. **Establish boundaries**: Communicate your limits to others and be clear about what you can handle. 4. **Practice relaxation techniques**: Engage in deep breathing exercises, meditation, or listen to calming music to relax your mind and body. 5. **Change your environment**: If your current surroundings are overwhelming, find a quiet and peaceful place where you can unwind. 6. **Take breaks**: Make sure to take regular breaks when needed and prioritize getting enough rest. 7. **Talk to someone**: Share your feelings with a trusted family member or friend who can provide support and understanding. 8. **Engage in physical activity**: Incorporate physical activities like walking, gardening, or dancing into your routine to reduce stress. 9. **Seek professional help**: If stress becomes overwhelming, don't hesitate to reach out to a healthcare professional for guidance. 10. **Use humor**: Find moments of laughter and use humor as a tool to manage stress and uplift your mood. 11. **Set realistic goals**: Focus on what you can accomplish each day and avoid putting excessive pressure on yourself. 12. **Establish a routine**: Having a structured daily schedule can help reduce stress and increase productivity. 13. **Pursue hobbies**: Make time for activities you enjoy, even if it's just for a short while. 14. **Get enough sleep**: Avoid consuming alcohol and caffeine in the evening to improve the quality of your sleep.   These strategies can effectively help you manage your emotions and reduce stress as a caregiver.  Source: (webpage) Reducing Stress , (document) By Us For Us_Managing triggers.txt , (webpage) Caregiver Stress , (webpage) Alzheimer’s Caregiving: Caring for Yourself , (webpage) Tips for Daily Life , (document) Alzheimer's Society_Caring for person with dementia practical guide.txt |
| How do I take breaks for myself? | To prioritize self-care, here are some strategies you can try:   1. **Schedule pleasant activities**: Make time for enjoyable activities every day, such as listening to music, meeting friends, or getting a massage. 2. **Practice relaxation techniques**: Regularly engage in relaxation methods like slow breathing exercises to manage stress. 3. **Distract yourself**: Shift your focus away from stressful situations by doing another task, pursuing a hobby, or practicing grounding techniques. 4. **Seek support**: Ask family, friends, or neighbors to temporarily take over caregiving responsibilities so you can take a break. 5. **Adjust your mindset**: Challenge unrealistic expectations and beliefs to reduce stress and improve your emotional well-being.   These steps can help you better cope with your emotions and stress while taking care of yourself.  Source: (document) PDC30 Guidebook Module 1.3.docx | - **Remember to take regular breaks**: It's crucial to take regular breaks from caregiving to maintain your own well-being. This will help you cope better and provide better support to the person you are caring for. - **Don't hesitate to ask for help**: Reach out to family, friends, or professional carers to get some assistance and take some time for yourself. - **Engage in activities you enjoy**: Utilize your break time to do something you love, like meeting a friend for coffee, pursuing a hobby, or simply relaxing. - **Plan your breaks**: Schedule breaks from your responsibilities, especially in long-term caregiving situations, and make sure you have a safe space to unwind. - **Consider respite care services**: Explore the option of using respite care services to take a break. This can be for a few hours, days, or even weeks. - **Practice relaxation techniques**: Engage in activities such as yoga, meditation, or deep breathing exercises to help reduce stress and rejuvenate yourself. - **Socialize and connect**: Spend time with friends or join a support group to share your experiences and reduce feelings of isolation. - **Set realistic goals**: Focus on what you can accomplish and don't put too much pressure on yourself to do everything at once. Take breaks when tasks become too challenging.   Source: (document) Alzheimer's Society_Carers look after yourself.txt , (document) Word Health Organization iSupport manual.txt , (webpage) Alzheimer’s Caregiving: Caring for Yourself , (webpage) Reducing Stress , (document) By Us For Us_Support matters.txt , (document) Alzheimer's Society_Understanding & supporting person with dementia.txt , (document) Alzheimer's Society_Caring for person with dementia practical guide.txt , (document) By Us For Us_Enhancing social connections.txt , (webpage) Tips for Daily Life |
| I can’t control my anger | To control your anger effectively, consider the following strategies:   1. **Walk Away**: If the situation allows, walking away can help both you and the other person calm down. 2. **Switch Attention**: Redirect your focus to something neutral or positive to avoid escalating conflicts. 3. **Practice Tolerance**: Understand that getting upset frequently is not beneficial. Strive to remain calm even when provoked. 4. **Take Breaks**: If you find yourself getting annoyed, take a break or switch your attention to another task. 5. **Avoid Confrontation**: Avoid arguing or trying to convince the other person, as this can escalate the situation. 6. **Self-Distraction**: Engage in another activity, practice relaxation techniques, or find a hobby to distract yourself. 7. **Slow Breathing**: Use slow breathing techniques to calm down. For example, inhale for 4 seconds, hold for 4 seconds, and exhale for 6 seconds. 8. **Positive Thinking**: Focus on positive thoughts and try to see the situation from a different perspective. 9. **Seek Support**: Talk to friends, family, or professionals to help manage your emotions and stress.   By implementing these strategies, you can manage your anger more effectively and maintain a calmer demeanor.  Source: (document) PDC30 Guidebook Module 2.4.docx , (document) PDC30 Guidebook Module 1.3.docx , (document) PDC30 Guidebook Module 1.5.docx | (second search)^a^  Here are some tips to manage anger effectively:   - **Practice deep breathing**: This helps oxygen reach your brain, which can be especially important if you are not physically active. - **Enter a quiet place with softer lighting** or close your eyes to help calm down. - **Meditate** or listen to soothing/calming music depending on your mood and personality. - **Participate in something that will distract you**: Engaging in a different activity can help shift your focus. - **Take a breath of fresh air and calm down**: Counting to 10 can also help. - **Share your feelings with others**: Talking to a willing listener can help ease your frustrations. - **Avoid situations and things that trigger a negative response**: Try to remove yourself from stressful situations and control your environment as best you can. - **Get plenty of rest to avoid fatigue**: Fatigue can exacerbate feelings of anger and frustration. - **Use relaxation techniques**: Music, exercise, reading, deep breathing, and meditation can all help reduce anxiety. - **Take breaks when you need to**: Also, get plenty of rest to help conserve your energy. - **Change your environment**: If you are in an environment that has too much stimulation causing you stress, find a quiet place where you can relax and regroup. - **Talk with a trusted family member or friend about how you’re feeling**: Sharing your thoughts and feelings can provide relief. - **Take a “timeout” when frustrated**: Take deep breaths, count to 10, or leave the room for a few minutes.   These strategies can help you manage your anger more effectively and improve your overall emotional health.  Source: (document) By Us For Us_Managing triggers.txt , (webpage) Reducing Stress , (webpage) Taking Care of Yourself , (webpage) What Are Frontotemporal Disorders? Causes, Symptoms, and Treatment |
| This is so tough. I’m afraid I can’t continue. | Being a caregiver can be incredibly challenging and overwhelming. It's normal to feel physically and emotionally exhausted due to the constant demands and responsibilities. Sometimes, you may even doubt your abilities and feel stressed. Witnessing your loved one's decline and the fear of making mistakes can also contribute to feelings of frustration and helplessness. Remember to seek support, take breaks, and remind yourself of your motivations and the positive moments. These strategies can help you manage these emotions effectively.  Source: (document) PDC30 Guidebook Module 1.3.docx , (document) PDC30 Guidebook Module 1.5.docx | (second search)^a^  It seems like you're going through a lot of stress and frustration, which can make caregiving challenging. It's important to understand that caregiving can be overwhelming, and it's okay to ask for help and take breaks when needed. You may also be feeling isolated or unsupported, which can make things even harder. Remember to be kind to yourself and focus on what you can do today. If necessary, reach out to friends, family, or professionals for support in managing the responsibilities.  Source: (document) By Us For Us_Support matters.txt , (document) Word Health Organization iSupport manual.txt , (webpage) Helping Family and Friends , (webpage) Tips for Daily Life , (document) By Us For Us_Role health wellbeing.txt |
| I am mad with myself. I made so many mistakes. | It's completely normal to feel frustrated with yourself when you make mistakes, especially in challenging situations like caregiving. Here are some insights and suggestions to help you manage these emotions:   1. **Recognize Your Emotions**: Understand that caregiving is a tough job and making mistakes is a natural part of the learning process. Many caregivers have made mistakes but continue to grow and improve. 2. **Be Kind to Yourself**: Show yourself compassion and remember that the behaviors of your loved one are a result of the disease, not personal attacks against you. 3. **Set Realistic Expectations**: Avoid putting pressure on yourself to be perfect. Unrealistic beliefs can lead to burnout and self-blame, making it harder to recover. 4. **Take Breaks**: Make sure to schedule personal time for yourself to prevent physical and emotional health issues in the long run. 5. **Seek Support**: Don't hesitate to ask for help from neighbors, friends, and family. Many people are willing to lend a hand, which can lighten your load. 6. **Practice Positive Self-Talk**: Use positive thinking and humor to transform difficult situations into more pleasant ones. This can help improve your mood and better manage stress.   By acknowledging your feelings and taking steps to manage them, you can minimize the impact of negative emotions and focus on positive actions.  Source: (document) PDC30 Guidebook Module 1.5.docx , (document) PDC30 Guidebook Module 1.1.docx , (document) PDC30 Guidebook Module 1.3.docx | It's completely normal to feel frustrated or angry when things don't go as planned. Here are some insights and suggestions to help you manage these feelings:   - **Understanding Your Emotions**: Recognize that these emotions are a natural response to the situation. - **Being Kind to Yourself**: Have patience with yourself and focus on what you can do today. - **Setting Realistic Expectations**: Understand that some tasks may be too difficult to complete, and that's okay. - **Taking Breaks**: If something becomes too difficult, take a break and try again later. - **Seeking Support**: Don't hesitate to ask for help from friends, family, or professionals. - **Positive Self-Talk**: Remind yourself that you are doing the best you can and that it's okay to make mistakes.   By acknowledging your feelings and taking steps to manage them, you can reduce the impact of negative emotions and focus on positive actions.  Source: (document) Alzheimer's Society_Carers look after yourself.txt , (document) By Us For Us_Managing triggers.txt , (webpage) Tips for Daily Life , (webpage) Taking Care of Yourself , (document) By Us For Us_Memory workout.txt , (document) By Us For Us_Support matters.txt , (document) Word Health Organization iSupport manual.txt , (webpage) Alzheimer’s Caregiving: Caring for Yourself |
| I am so frustrated I can’t control my emotions. I would even take out my irritation on her. | When you're feeling frustrated and struggling to control your emotions, and you have the urge to take it out on someone, try these strategies:   - **Take a Break**: If you're really annoyed, step away or shift your focus to something else to calm down before things escalate. - **Practice Emotional Control**: Tolerance and emotional control are key. Staying calm, even when provoked, helps prevent harm to both you and your loved one. Deep breathing may help. - **Walk Away**: If the situation becomes physically aggressive, walking away can help both parties calm down and avoid further escalation. - **Avoid Nagging**: Constant complaints or anger can be seen as hostility and may provoke your loved one even more, leading to more frustration. - **Self-Distraction**: Engage in self-distraction by redirecting your attention to something neutral or positive to avoid confrontation and reduce conflicts. Putting on headphones and listening to music may help. - **Use Humor and Positive Thinking**: Try to turn challenging events into amusing and positive experiences by using humor and maintaining a flexible perspective. - **Grounding Techniques**: Shift your attention by naming a few things in your surroundings that you can see, touch, hear, or smell, and focus on these objects instead of the difficult situation. - **Gentle Communication**: Communicate gently and avoid arguing. For example, if your loved one gets lost, speak to them in a soft voice and offer gentle guidance. - **Mindset Adjustment**: Adjust your mindset by focusing on positive thoughts and finding something enjoyable to do each day. - **Seek Support**: Don't hesitate to ask for help from neighbors, friends, and family to lighten your load and alleviate some of the burdens. - **Focus on the Process of Doing Things Together**, not the results.   These strategies can help you manage your emotions and improve your interactions with your loved one.  Source: (document) PDC30 Guidebook Module 1.3.docx , (document) PDC30 Guidebook Module 1.5.docx , (document) PDC30 Guidebook Module 2.4.docx , (document) PDC30 Guidebook Module 1.4.docx , (document) PDC30 Guidebook Module 2.3.docx | To maintain control over your emotions and prevent venting your frustration on others, consider the following techniques:   - **Practice Deep Breathing**: This will help calm your mind and body by increasing oxygen flow to your brain. - **Find a Quiet Place**: Retreat to a serene environment with softer lighting or close your eyes to reduce stimulation. - **Meditate**: Engaging in meditation can help center your thoughts and alleviate stress. - **Listen to Soothing Music**: Music has a calming effect and can divert your attention from negative emotions. - **Participate in Distracting Activities**: Engage in activities that divert your attention from stressors. - **Share Your Feelings**: Open up to a trusted friend or family member about your emotions. - **Take Breaks**: Allow yourself to step back and take breaks when needed to prevent feeling overwhelmed. - **Positive Self-Talk**: Remind yourself that you are doing your best and it's okay not to be perfect. - **Avoid Arguing**: Try to avoid arguments or confrontations with the person you are caring for, as this can escalate emotions. - **Seek Support**: Don't hesitate to ask for help from neighbors, friends, and family to lighten your load.   By implementing these strategies, you can effectively manage your emotions and prevent venting your frustration on others.  Source: (document) By Us For Us_Managing triggers.txt , (webpage) Taking Care of Yourself , (webpage) Reducing Stress , (webpage) Alzheimer’s Caregiving: Caring for Yourself , (document) Word Health Organization iSupport manual.txt |
| My Mom sits there all day. I cannot get her to do anything. What am I supposed to do? | - **Recognize the reasons**: It's important to understand that apathy and difficulty initiating behaviors are common in dementia. This understanding can help you approach the situation with more empathy. - **Take the lead**: Sometimes, simply taking your mom's hand and initiating the activity can be helpful. Use encouraging words to motivate her. - **Plan daily activities**: Creating a schedule of activities throughout the day can provide structure and reduce inactivity, which can contribute to a more rapid decline. - **Engage in joint activities**: Include activities that both you and your mom can enjoy, such as going for a walk, playing word games, or drawing. - **Simplify choices**: Minimize the number of choices to avoid overwhelming her, as this can lead to withdrawal from participation. - **Be patient and gentle**: Interact with her in a patient and gentle manner to avoid causing frustration or agitation. - **Create a comfortable environment**: Ensure that the environment is comfortable and free from distractions that might cause agitation. - **Use positive reinforcement**: Encourage and praise her for any participation, no matter how small, to build her confidence and willingness to engage.   These steps can help encourage your mom to be more active and engaged throughout the day.  Source: (document) PDC30 Guidebook Module 1.5.docx , (document) PDC30 Guidebook Module 2.2.docx , (document) PDC30 Guidebook Module 1.2.docx , (document) PDC30 Guidebook Module 2.3.docx | - **Engage the person in an activity**. Sometimes, the person may just be feeling bored and in need of something to do. It can be helpful to provide structure and involve them in a pleasant activity. - **Involve the person through conversation**. While you're doing tasks like cooking dinner, try talking to the person about what you're doing. Even if they can't respond, they may still benefit from the communication. - **Substitute an activity for a behavior**. If the person with dementia is idle, you can offer a simple task like wiping a table or tapping to music. - **Help get the activity started**. Many people with dementia still have the energy and desire to do things, but they may struggle with organizing, planning, initiating, and completing tasks. Offering support and guidance can help them get started. - **Offer support and supervision**. You may need to demonstrate how to perform the activity and provide clear, easy-to-follow steps. - **Concentrate on the process, not the result**. What matters is that you're able to spend time together and that the person feels a sense of accomplishment. - **Be flexible**. If the person insists they don't want to do something, it could be because they can't do it or they're afraid. It's important not to force it. If they want to do it in a different way, allow it and make adjustments later if needed. - **Break activities into simple, easy-to-follow steps**. Focus on one task at a time. Giving too many directions at once can be overwhelming.   Source: (webpage) Repetition , (webpage) Activities , (webpage) 50 Activities |
| How can I allow my husband to wander safely? | - **Accompany him on walks**: Consider walking with your husband to spend more quality time together and ensure his safety. - **Use gentle communication**: If he becomes disoriented, speak to him in a calm and soothing manner to guide him back home. - **Identification and tracking**: Make sure he wears an identification tag, carries a letter explaining his condition, has emergency contact numbers, and uses a GPS tracking device. - **Engage in structured activities**: Plan activities that align with his wandering behavior, such as regular walks, which can help manage restlessness and provide physical exercise. - **Stick to familiar surroundings**: Opt for activities close to home to provide a sense of security and make it easier to return home if needed. - **Implement safety measures**: Install safety knob covers and store away hazardous items to prevent accidents at home.   Source: (document) PDC30 Guidebook Module 1.3.docx , (document) PDC30 Guidebook Module 1.5.docx , (document) PDC30 Guidebook Module 2.3.docx , (document) PDC30 Guidebook Module 1.4.docx , (document) PDC30 Guidebook Module 2.5.docx | To ensure the safety of your husband while allowing him to wander, you can try the following strategies:   1. **Walk Together**: Accompany him on walks to spend quality time together and ensure his safety. 2. **Identification**: Make sure he wears an identification tag, carries a letter explaining his condition, and has emergency contact numbers. 3. **GPS Tracking**: Consider using a GPS tracking device to easily locate him if he wanders off. 4. **Routine Walks**: Incorporate regular walks into his daily routine to manage restlessness and provide exercise. 5. **Safety Measures at Home**: Install safety knob covers and remove any potentially dangerous items to prevent accidents at home. 6. **Home Security**: Take steps to secure your home, such as camouflaging doors and door knobs or using a bell or curtain that makes noise when someone uses the door. 7. **Photograph**: Keep an up-to-date photograph of him in case he gets lost and you need to seek help from others. 8. **Calm Communication**: If he does get lost, speak to him gently and calmly to guide him back home.   By implementing these strategies, you can better manage the situation while ensuring his dignity and comfort.  Source: (document) Word Health Organization iSupport manual.txt |
| How do I get my Dad to enjoy his meals? | - **Serve small but frequent meals**: Small but frequent meals throughout the day can make it easier for him to handle and may encourage him to eat more. - **Eat together**: Try eating with your dad and matching your pace to his. This can make the meal more enjoyable for both of you and may encourage him to eat more. - **Offer variety**: Serve food with a variety of colors, tastes and textures to make food more appealing. - **Serve finger foods**: If your dad has difficulties using utensils, try cutting the food into manageable portions that can be eaten with fingers. This can make it easier for him to eat. - **Encourage self-feeding**: You can gently touch your dad's lip with a spoon containing food to help initiate the eating action. - **Limit distractions**: Creating a calm and well-lit environment during mealtimes can make them more pleasant and less confusing for your dad. - **Be flexible with food preferences**: Offer foods that your dad likes and be willing to adjust based on his changing tastes. - **Offer one food item at a time**: Minimize choices to avoid overwhelming your dad and make the decision easier for him. - **Use adaptive utensils**: Consider using utensils with bigger or special handles to make eating easier for your dad.   Source: (document) PDC30 Guidebook Module 2.2.docx , (document) PDC30 Guidebook Module 1.5.docx , (document) PDC30 Guidebook Module 2.4.docx , (document) PDC30 Guidebook Module 2.5.docx , (document) PDC30 Guidebook Module 1.2.docx | - **Establish a consistent routine for serving meals**. This helps create a sense of structure. - **Offer familiar and preferred foods** to make meals more appealing. - **Use mealtime as an opportunity to discuss enjoyable topics**. This helps create a positive atmosphere. - **Minimize distractions by keeping the eating area quiet**. Turn off the TV and radio. - **Ensure that the food is easy to eat** by cutting it into small pieces and making it soft. - **Present one food item at a time and avoid rushing the meal**. This prevents overwhelming the person. - **Experiment with different types of food and drink**. Vary the temperatures and textures to find what they enjoy. - **Provide gentle reminders to eat and remind them of the food**. This encourages eating. - **Make mealtimes an opportunity for activity and social interaction**. Engage the person in conversation and other activities. - **Encourage their involvement in meal preparation**. Let them set the table or help with food preparation. - **Keep healthy snacks within reach** for them to enjoy when desired. - **Make the food visually and aromatically appealing**. Use different colors, tastes, and smells. - **Avoid overloading the plate with too much food**. Small and regular portions are often more manageable. - **Promote physical activity**, as it can increase appetite.   Source: (document) National Institute on Aging website on Six Tips To Make Mealtimes Easier for People With Alzheimer’s Disease.txt , (document) Alzheimer's Society_Factsheet eating & drinking.txt , (document) Alzheimer's Society_Caring for person with dementia practical guide.txt |
| My Mom refuses to change her clothes. What should I do? | If your mom is refusing to change her clothes, here are some strategies you can consider:   1. **Minimize Choices**: Offer her fewer options to avoid overwhelming her. For example, you can pick one outfit and say, "Hey, this looks nice. You look great in it. Will you wear this at the dinner tonight?" This approach provides an element of choice without overwhelming her. 2. **Be Patient and Gentle**: If she refuses initially, try to ignore the situation, wait, do something else, and then try again later. This can sometimes turn things around. 3. **Provide Support and Understanding**: Understand that she may feel embarrassed or uncomfortable. Avoid making her feel more embarrassed by being sensitive to her feelings. 4. **Simplify Instructions**: Break down the task into smaller, manageable steps and guide her through each step with clear, concrete instructions. 5. **Create a Positive Environment**: Use positive reinforcement and create a supportive environment. For example, you can reward her with something she likes after she changes her clothes.   These steps can help manage the situation more effectively while maintaining her dignity and comfort.  Source: (document) PDC30 Guidebook Module 2.2.docx , (document) PDC30 Guidebook Module 2.4.docx , (document) PDC30 Guidebook Module 1.5.docx , (document) PDC30 Guidebook Module 1.2.docx , (document) PDC30 Guidebook Module 1.1.docx | If your mom is refusing to change her clothes, there are several strategies you can try:   1. **Remain calm and find a way around it**: Think about her routine before dementia and encourage her to maintain cleanliness. 2. **Remove dirty clothing and replace with clean clothing**: Do this when she is bathing, sleeping, or showering. Having multiple sets of the same items can be helpful if she insists on wearing the same clothes. 3. **Encourage her to change for specific occasions**: For example, when someone is visiting or when she is going out. 4. **Lay out clothes in the order they should be put on**: Hand her one item at a time or provide step-by-step dressing instructions. 5. **Reduce the number of choices**: Keep only one or two outfits in the closet or dresser to avoid overwhelming her. 6. **Buy multiple sets of the same clothes**: If she wants to wear the same clothing every day, having duplicates can be useful. 7. **Choose comfortable and simple clothing**: Opt for loose-fitting, comfortable clothing with easy fastenings like Velcro® instead of buttons or zippers. 8. **Check for physical problems**: Ensure there are no physical issues that make it difficult for her to change clothes. 9. **Be flexible with timing**: If she refuses to change at a particular time, try again later.   These strategies can help make the process smoother and less stressful for both of you.  Source: (document) Alzheimer's Society_Washing & bathing.txt , (webpage) Bathing, Dressing, and Grooming: Alzheimer’s Caregiving Tips , (webpage) Dressing and Grooming , (document) Alzheimer's Society_Supporting person washing & dressing.txt |
| My Dad refuses to bathe. What should I do? | - **Take a break, try again later**: Sometimes, taking a break and trying again later can be helpful. - **Encourage gently and offer rewards**: Encouraging your dad gently and offering rewards after the bath can motivate him to comply. - **Understand the reasons for refusal**: Your dad may feel embarrassed, want privacy, or not see the need for a bath. Understanding these reasons can help address his concerns. - **Be flexible and patient**: Avoid sticking to a rigid schedule and be prepared to adapt to changing circumstances. - **Create a comfortable environment**: Ensure the bathing area is safe and comfortable, such as using a non-slippery floor in the shower or giving a sponge bath. - **Minimize choices and be direct**: Instead of giving multiple options, make straightforward statements like "It's time to bathe." - **Distract and redirect attention**: If ignoring doesn't work, try distracting your dad with something interesting or asking for his help with a task. - **Respect his dignity and autonomy**: Be considerate of his feelings and avoid making him feel embarrassed.   Source: (document) PDC30 Guidebook Module 2.4.docx , (document) PDC30 Guidebook Module 1.5.docx , (document) PDC30 Guidebook Module 1.2.docx , (document) PDC30 Guidebook Module 2.2.docx , (document) PDC30 Guidebook Module 2.5.docx , (document) PDC30 Guidebook Module 1.1.docx | If your Dad refuses to bathe, you can try the following strategies:   1. **Timing and Phrasing**: Consider the timing of your request and how you phrase it. He may refuse to wash when you suggest it, but might decide to wash himself later in the day. Try to be flexible. 2. **Provide a Reason**: Persuade him to wash by giving him a reason, such as if he is going out or expecting visitors. 3. **Health Concerns**: Speak with the GP about any health concerns you have regarding his hygiene. 4. **External Carers**: Consider arranging external carers to carry out personal care. A needs assessment with social services can help determine what care and support might be available. 5. **Sponge Bath**: If he refuses a bath or shower, try a sponge bath at least twice a week. You can wash him with a wet sponge or cloth without him getting into a bath or under a running shower. 6. **Distraction**: If he becomes aggressive when you suggest washing, change the subject or distract him and try again later. 7. **Comfort and Privacy**: Make the experience as pleasant and relaxed as possible. Ensure the room is warm and consider using nice-smelling bubble bath or relaxing music. If he seems shy or embarrassed, uncover only the part of his body that you are washing. 8. **Choices and Control**: Give him choices, such as asking if he would like to bathe now or in 15 minutes, or take a bath or a shower. Try saying “Let’s wash up,” instead of “Let’s take a bath”. 9. **Familiar Person**: Try having a familiar person of the same sex help with bathing if that is more comfortable for him.   These strategies can help make the bathing process easier and more comfortable for both of you.  Source: (document) Alzheimer's Society_Washing & bathing.txt , (document) Alzheimer's Society_Caring for person with dementia practical guide.txt , (webpage) Bathing |
| I am worried about my husband continuing to drive. How do I get him to stop? | To address your concern about your husband continuing to drive, consider the following strategies:   - **Be Kind and Patient**: Speak to him in a gentle and calm manner, as he may respond better to a gentle approach. Avoid arguing or trying to reason with him if he becomes agitated. - **Consider His Emotions**: Understand that he may feel his independence is being threatened, so approach the topic with warmth and respect. - **Highlight Safety Concerns**: Explain the potential dangers of driving with dementia, such as impaired eye-hand coordination and visuo-spatial-motor skills. - **Distraction by Offering Alternatives**: Engage him in activities that he enjoys and can be done together, such as walking, to replace the need for driving while you can also watch over him. - **Safety Measures**: If he insists on going out, make sure he has an identification tag, carries a letter explaining his condition, and uses a GPS tracking device for safety. - **Seek Professional Advice**: Consult with a healthcare professional to discuss the best approach and possibly get a formal assessment of his driving abilities.   These steps can help manage the situation more effectively while maintaining his dignity and comfort.  Source: (document) PDC30 Guidebook Module 1.5.docx , (document) PDC30 Guidebook Module 1.3.docx , (document) PDC30 Guidebook Module 1.1.docx | - **Initiate a conversation to express your concerns**: Emphasize the positive and offer alternatives. - **Address resistance while reaffirming your unconditional love and support**: Appeal to the person's sense of responsibility. - **Involve your physician**: Request your physician to advise the person not to drive. The physician can write a letter or prescription stating that the person with Alzheimer’s must not drive. - **Consider an evaluation by an objective third party**: This can provide a more objective understanding of the current impact of the disease on driving capacity. - **Be patient and firm**: Demonstrate understanding and empathy. Acknowledge the pain of this change and appeal to the person's desire to act responsibly. - **Ask a respected family authority figure or your attorney to reinforce the message**: This can help in situations where the person is resistant. - **As a last resort, take away the car keys, disable the car, or consider selling the car**: Ensure to provide safe, reliable alternative transportation. - **Plan ahead and discuss when driving should stop**: Use a positive approach when communicating driving safety with people living with dementia. - **Talk about the practical consequences**: Point out the financial cost if they were to have an accident or the personal cost if they were to injure someone. - **Involve family and close friends in the plan**: This can help in confronting resistance and preparing for the future. - **Consider signing a driving contract**: When the person is still in the early stage of Alzheimer’s, ask them to sign a driving contract that gives you permission to help them stop driving when the time comes.   Source: (webpage) Dementia and Driving , (document) By Us For Us_Living safely.txt |
| My Mom accuses me of stealing her stuff. What should I do? | - **Avoid arguing**: It's best to avoid getting into arguments with her. Arguing will only make her more upset and defensive. - **Take a break**: Sometimes, taking a break from the situation can help. You can step out to buy something and by the time you return, she might have forgotten about the issue. - **Distract her**: Suggesting a favorite activity or pastime can help divert her attention and shift her focus. - **Understand her reality**: It's important to respect her altered reality and avoid directly confronting her. Trying to convince her otherwise may only lead to more frustration. - **Use therapeutic lying**: If necessary, it can be helpful to use white lies to calm her down. For example, you can say that the items are being cleaned or repaired.   Source: (document) PDC30 Guidebook Module 2.4.docx | - **Don't take offense**. Listen attentively to what is troubling the person, and make an effort to comprehend their reality. Then, provide reassurance and let them know that you genuinely care. - **Avoid arguing or trying to convince**. Allow the individual to express their ideas and validate their opinions. - **Offer a simple answer**. Share your thoughts with simplicity, avoiding lengthy explanations or justifications that may overwhelm them. - **Redirect their attention to another activity**. Engage the person in a different task or ask for their assistance with a chore. - **Provide duplicates of lost items**. If the person frequently searches for a specific item, ensure that there are multiple copies available. For instance, if they often misplace their wallet, consider purchasing two of the same kind. - **Help others understand changing behaviors**. Educate family members and caregivers about the fact that suspicions and false accusations are a result of the disease and not a reflection of their character.   Source: (webpage) Suspicions and Delusions |
| My Dad is scolding me every day. I cannot take it anymore! | - **Remember to take breaks**. If things get too overwhelming, it's important to step back and give yourself some time to recharge. - **Open up to someone**. Share your feelings with a trusted family member or friend who can provide support. - **Create a peaceful environment**. Minimize noise and distractions to help reduce stress for your dad. - **Focus on emotions, not just facts**. Try to understand the feelings behind the words, rather than getting caught up in the details. - **Seek out support**. Consider joining a support group or speaking with a counselor to help manage your emotional well-being. - **Understanding the cause**. Dementia changes the person. Don’t take things personal.   Source: (document) PDC30 Guidebook Module 2.4.docx | (second search)^a^   - **Understanding the cause**: It's important to recognize that behavior changes, such as aggression, can be a result of dementia or a reaction to the disease. - **Avoid self-blame**: Remember that these behaviors are caused by the disease and are not a reflection of your worth as a caregiver. - **Maintain a calm demeanor**: Try to stay calm and avoid confrontations or arguments about facts. - **Redirect attention**: Engage your dad in a different activity to divert his attention from the behavior. - **Seek support**: Consider seeking help from a counselor if caregiving becomes overwhelming for you. - **Take care of yourself**: Remember to prioritize your own well-being and manage your stress levels. Take breaks when necessary.   Source: (document) Word Health Organization iSupport manual.txt , (webpage) Alzheimer’s Caregiving: Caring for Yourself , (webpage) Treatments for Behavior , (webpage) Taking Care of Yourself , (document) By Us For Us_Support matters.txt |
| Lately I find that my Mom starts to wet her underwear. She doesn’t want to talk about it. What should I do? | - **Show empathy and understanding**: It's important to understand that your mom may feel embarrassed and ashamed about her incontinence. Avoid reacting with shock or embarrassment, as this can make her feel worse. - **Seek professional guidance**: Consider reaching out to a nursing professional for advice on alternative products and methods to manage incontinence. There are discreet options available that can help address this issue. - **Handle the situation sensitively**: If your mom is unwilling to discuss the issue directly, avoid confronting her. Instead, try to address the problem in a sensitive and non-embarrassing manner. - **Offer practical solutions**: Introduce practical solutions such as using clothing items that are easier to manage and reducing choices to avoid overwhelming her. If needed, smaller absorbent pads may be more acceptable to your mom than diapers. - **Create a supportive environment**: Ensure that the environment is well-lit and easy to navigate, which can help prevent accidents. For example, adding labels or using contrasting colors in the bathroom can make it easier for her to find the toilet.   These steps can help you effectively manage the situation while preserving your mom's dignity and comfort.  Source: (document) PDC30 Guidebook Module 1.2.docx , (document) PDC30 Guidebook Module 1.1.docx , (document) PDC30 Guidebook Module 1.5.docx , (document) PDC30 Guidebook Module 2.2.docx , (document) PDC30 Guidebook Module 2.1.docx , (document) PDC30 Guidebook Module 2.5.docx | - **Stay Calm and Reassure**: Accidents happen. Be understanding when they occur. Stay calm and reassure the person if she is upset. - **Use Incontinence Supplies**: Consider using incontinence supplies such as adult disposable briefs or underwear, bed protectors, and waterproof mattress covers. These items can be purchased at drugstores and medical supply stores. - **Maintain Hygiene**: If an accident occurs, ensure that she washes as soon as possible with mild soap and warm water, dries carefully, and puts on clean clothes. - **Set a Schedule**: Keep a record of how much food and fluid she takes in and how often she goes to the bathroom. Use this information to make a schedule of when she needs to go to the bathroom. - **Seek Professional Help**: If you find feelings about incontinence difficult to handle, talk things through with a health professional such as a GP, community nurse, or continence adviser. - **Be Matter-of-Fact**: Approach the situation with a matter-of-fact attitude and avoid appearing angry or upset. This can help maintain her dignity and self-esteem. - **Use Protective Products**: Consider using absorbent and protective products like incontinence pads or pull-up pants to soak up urine. - **Limit Fluids Before Bedtime**: Limit fluids at least two hours before bedtime to reduce nighttime accidents, but ensure she gets adequate fluids during the day to avoid dehydration.   Source: (webpage) Alzheimer’s Disease: Common Medical Problems , (document) Alzheimer's Society_Caring for person with dementia practical guide.txt , (document) Alzheimer's Society_Factsheet continence & using toilet.txt , (webpage) For Kids , (document) Word Health Organization iSupport manual.txt , (webpage) Late-Stage Caregiving |

Note. “(webpage)” with a shaded background indicates a webpage from National Institute on Aging, US. Otherwise the (webpage) is from Alzheimer’s Association. Botpress cannot scan organizational logo and hence can only index the webpage by its title but not the name of the organization. There is currently no technique to deal with this citation problem. To enumerate the bullet points, the chatbots switched between numerals and the dot symbol; these were kept as originally generated.

^a^“(second search)” means that the first search ended with no results; a second attempt was needed to generate this answer.
