# Supplementary material for: The PDC30 Chatbot—Development of a Psychoeducational Resource on Dementia Caregiving Among Family Caregivers: Mixed Methods Acceptability Study
Source: JMIR Aging. 2025 Jan 6;8:e63715. doi: 10.2196/63715 (PMC11758934; doi:10.2196/63715)
Supplement: Multimedia Appendix 2 [file aging_v8i1e63715_app2.docx]

**Responses provided by ChatGPT**

| Modified questions to highlight dementia context^a^ | ChatGPT responses |
| --- | --- |
| What is dementia? | Dementia is a general term for a decline in cognitive function severe enough to interfere with daily life and activities. It is not a specific disease but rather a syndrome that may result from a variety of underlying diseases and conditions. Dementia affects memory, thinking, language, problem-solving, and other cognitive abilities, as well as behavior and emotions.  The most common cause of dementia is Alzheimer's disease, which accounts for 60-80% of cases. Other causes include vascular dementia, which results from conditions that block or reduce blood flow to the brain, Lewy body dementia, frontotemporal dementia, and mixed dementia, which is a combination of two or more types.  Symptoms of dementia can vary widely but often include:   - Memory loss - Difficulty communicating or finding words - Difficulty with complex tasks and problem-solving - Confusion and disorientation - Changes in mood and behavior - Difficulty with coordination and motor functions - Withdrawal from work or social activities   Dementia is typically progressive, meaning symptoms gradually worsen over time. While there is no cure for most types of dementia, certain medications and therapies can help manage symptoms and improve quality of life. Early diagnosis and intervention can also provide important benefits. |
| I cannot accept the idea that my Mom has dementia and may leave me one day. | I'm really sorry to hear about your Mom's condition. Dealing with a loved one's dementia can be incredibly challenging and emotional. If you're looking for information or resources, here are a few suggestions that might help:   1. **Understanding Dementia**: Learning more about dementia can help you understand what your mom is going through and what to expect. Websites like the Alzheimer's Association (alz.org) provide comprehensive information about the different types of dementia, symptoms, stages, and treatments. 2. **Support Groups**: Connecting with others who are going through similar experiences can be incredibly comforting. There are many online and local support groups for families and caregivers of people with dementia. The Alzheimer's Association offers support groups and community forums. 3. **Caregiving Resources**: There are numerous resources available to help caregivers manage the day-to-day challenges of caring for someone with dementia. The Family Caregiver Alliance (caregiver.org) offers tips, guides, and support for caregivers. 4. **Professional Help**: Consulting with healthcare professionals who specialize in dementia care can provide you with personalized advice and support. This can include doctors, neurologists, geriatricians, and social workers. 5. **Emotional Support**: It's important to take care of your own mental health during this time. Consider speaking with a therapist or counselor who can help you process your feelings and develop coping strategies.   If you need more specific information or resources, please let me know how I can assist further. |
| How do I know what to expect, and how fast will things change **with dementia**? | Dementia is a progressive condition, and its course can vary significantly from person to person. While it is difficult to predict exactly how it will unfold for any individual, there are general stages and symptoms that are commonly observed. Here's what you might expect and some factors that can influence the progression:  **Stages of Dementia**  Dementia typically progresses through several stages:   1. *Mild (Early Stage):*    - Memory loss, particularly recent events    - Difficulty with problem-solving and complex tasks    - Changes in personality or mood    - Trouble with planning and organizing 2. *Moderate (Middle Stage):*    - Increased memory loss and confusion    - Difficulty recognizing friends and family    - Greater challenges with language and communication    - Wandering and getting lost    - Repetitive behavior    - Problems with personal care (e.g., bathing, dressing) 3. *Severe (Late Stage):*    - Significant memory loss    - Inability to recognize close family members    - Loss of physical abilities (e.g., walking, swallowing)    - Complete dependence on others for care    - Possible changes in behavior, such as aggression or agitation   **Rate of Progression**  The speed at which dementia progresses can vary widely based on several factors:   - *Type of Dementia:* Different types of dementia (e.g., Alzheimer's disease, vascular dementia, Lewy body dementia) have different progression rates. - *Age and Overall Health:* Younger individuals and those in better overall health may experience slower progression. - *Genetics:* Family history can play a role in how quickly dementia progresses. - *Lifestyle Factors:* Diet, exercise, social engagement, and mental stimulation can impact the progression. - *Medical Management:* Early diagnosis and appropriate medical interventions can sometimes slow the progression of symptoms.   **Managing Expectations**   - *Regular Medical Check-Ups:* Ongoing assessments by healthcare professionals can help monitor the progression and adjust care plans as needed. - *Education and Support:* Understanding the condition and joining support groups can help caregivers and patients cope better. - *Adaptations:* Making home modifications and establishing routines can help manage daily challenges. - *Legal and Financial Planning:* Early planning can help manage future needs and ensure that the wishes of the person with dementia are respected.   **Conclusion**  While it's challenging to predict exactly how dementia will progress in any individual, being informed and proactive can help manage the condition more effectively. Regular consultations with healthcare providers, staying informed about the condition, and having a strong support system are essential steps in navigating the journey with dementia. |
| What preparation should we make for the future **now that we know our relative has dementia**? | I'm sorry to hear about your relative's diagnosis. Preparing for the future when a loved one has dementia involves several steps to ensure their safety, well-being, and quality of life. Here are some key areas to consider:  **Medical Care**   1. *Regular Medical Checkups:* Schedule regular appointments with healthcare providers who specialize in dementia care. 2. *Medication Management:* Ensure that all medications are taken correctly. Consider using pill organizers or automated dispensers. 3. *Specialist Referrals:* Get referrals to neurologists, geriatricians, and other specialists as needed.   **Legal and Financial Planning**   1. *Power of Attorney:* Establish a durable power of attorney for healthcare and finances to ensure decisions can be made on their behalf. 2. *Advance Directives:* Create advance directives, including a living will, to outline medical care preferences. 3. *Estate Planning:* Update wills, trusts, and beneficiary designations to reflect the current situation. 4. *Financial Management:* Ensure bills are paid on time and finances are managed properly. Consider hiring a financial advisor with experience in elder care.   **Home Safety**   1. *Safety Assessment:* Conduct a home safety assessment to identify and mitigate potential hazards (e.g., secure rugs, install grab bars, ensure proper lighting). 2. *Wandering Prevention:* Install locks, alarms, or GPS tracking devices to prevent wandering. 3. *Emergency Preparedness:* Create a plan for emergencies, including a list of emergency contacts and medical information.   **Daily Care and Activities**   1. *Routine:* Establish a daily routine to provide structure and reduce anxiety. 2. *Engagement:* Engage them in activities they enjoy and can still participate in, such as puzzles, music, or light exercise. 3. *Professional Caregivers:* Consider hiring in-home caregivers or looking into adult day care programs.   **Emotional and Social Support**   1. *Support Groups:* Join support groups for both the person with dementia and their caregivers. 2. *Counseling:* Seek counseling for emotional support and coping strategies. 3. *Family Involvement:* Keep family members informed and involved in the care process.   **Long-Term Care Planning**   1. *Care Facilities:* Research long-term care options such as assisted living, memory care units, or nursing homes. 2. *Respite Care:* Look into respite care options to give primary caregivers a break.   **Education and Resources**   1. *Learn About Dementia:* Educate yourself and other family members about the progression of dementia and what to expect. 2. *Resources:* Utilize resources from organizations like the Alzheimer's Association or local support services.   By taking these steps, you can better prepare for the challenges ahead and ensure that your relative receives the best possible care and support. |
| Why is my wife acting like a different person **after having dementia**? | I'm really sorry to hear about what you're going through. Dementia can cause significant changes in a person's behavior, personality, and cognitive abilities. These changes can be very challenging for both the person with dementia and their loved ones. Here are a few reasons why your wife might be acting like a different person:   1. **Cognitive Decline**: Dementia affects the brain, leading to memory loss, confusion, and difficulty with thinking and reasoning. This can make it hard for your wife to recognize people, remember events, or understand her surroundings, which can result in behavior that seems out of character. 2. **Personality Changes**: Dementia can alter the parts of the brain that control personality and behavior. As a result, you might notice that your wife is acting differently than she used to. She might become more irritable, anxious, or even depressed, or she might show a lack of interest in activities she once enjoyed. 3. **Emotional Responses**: People with dementia often experience heightened emotions. They might become easily frustrated or agitated because they are aware of their own cognitive decline, or because they are struggling to communicate effectively. 4. **Hallucinations and Delusions**: Some types of dementia, such as Lewy body dementia or Alzheimer's disease, can cause hallucinations (seeing or hearing things that aren't there) and delusions (false beliefs). These can significantly alter a person’s behavior and how they interact with the world. 5. **Environmental Factors**: Changes in environment or routine can be particularly disorienting for someone with dementia. If your wife is in a new place or if there have been changes to her daily routine, this can exacerbate symptoms and lead to behavior that seems unusual. 6. **Physical Health**: Underlying physical health issues, such as infections, pain, or medication side effects, can also affect behavior. It's important to consider whether any new or worsening health problems might be contributing to the changes you're seeing. 7. **Communication Difficulties**: As dementia progresses, it often becomes more difficult for individuals to express their needs and feelings. This frustration can manifest in behavior that seems out of character.   It's important to consult with a healthcare professional who specializes in dementia. They can provide a more precise diagnosis and offer strategies to manage symptoms and improve quality of life. Support groups and counseling can also be very helpful for caregivers dealing with the emotional and practical challenges of caring for a loved one with dementia. |
| How can I communicate with my wife **who has dementia**? | Communicating with a loved one who has dementia can be challenging, but there are several strategies that can help you connect more effectively. Here are some tips:   1. **Be Patient and Calm:**    - Approach your wife with a calm and patient demeanor.    - Give her plenty of time to respond and avoid rushing her. 2. **Simplify Your Language:**    - Use short, simple sentences and speak slowly and clearly.    - Avoid complex questions; instead, ask yes/no questions or provide choices. 3. **Maintain Eye Contact:**    - Look into her eyes when you speak to her to help her focus on you.    - Use non-verbal cues, such as nodding and smiling, to reinforce your words. 4. **Minimize Distractions:**    - Choose a quiet environment with minimal background noise to have conversations.    - Turn off the TV or radio and ensure the room is well-lit. 5. **Use Visual Aids:**    - Show her pictures or objects related to what you’re talking about.    - Visual cues can help her understand and recall information better. 6. **Be Respectful:**    - Avoid talking down to her or treating her like a child.    - Acknowledge her feelings and validate her experiences, even if they seem confused. 7. **Repeat and Rephrase:**    - If she doesn’t understand something, try rephrasing it or repeating it slowly.    - Avoid saying “I just told you that” as it can be frustrating for her. 8. **Use Touch:**    - Gentle, reassuring touches like holding hands or a hug can convey warmth and comfort.    - Be mindful of her personal space and comfort level with physical contact. 9. **Stay Positive:**    - Focus on the positive aspects of your interactions.    - Celebrate small moments of connection and understanding. 10. **Encourage Involvement:**     - Engage her in familiar activities and routines that she enjoys.     - Encourage her to participate in conversations and activities to the best of her ability. 11. **Listen Actively:**     - Show that you are listening by nodding and giving verbal affirmations.     - Pay attention to her non-verbal cues, as they can provide insight into her feelings and needs. 12. **Seek Professional Support:**     - Consult with healthcare professionals for additional communication strategies tailored to your wife’s specific condition.     - Support groups and counseling can also provide emotional support and practical advice.   Remember that every person with dementia is unique, and what works one day may not work the next. Flexibility and empathy are key in adapting your communication approach to meet her evolving needs. |
| How can I manage my own feelings and stress **when caring for someone with dementia**? | Caring for someone with dementia can be incredibly challenging and stressful. Managing your own feelings and stress is crucial not only for your well-being but also for providing the best care possible. Here are some strategies that can help:  **1. Educate Yourself**  Understanding dementia can help you anticipate challenges and reduce frustration. Learn about the disease, its progression, and what to expect.  **2. Set Realistic Expectations**  Acknowledge that caring for someone with dementia is demanding and that you will have good days and bad days. Set realistic goals and don't strive for perfection.  **3. Seek Support**   - *Family and Friends:* Don't hesitate to ask for help from other family members or friends. - *Support Groups:* Join a support group for caregivers. Sharing experiences with others who understand can be incredibly comforting. - **Professional Help:** Consider counseling or therapy to help you process your feelings and develop coping strategies.   **4. Take Care of Your Health**   - *Physical Health:* Maintain a healthy diet, exercise regularly, and get enough sleep. - *Mental Health:* Practice mindfulness, meditation, or other relaxation techniques to help manage stress.   **5. Take Breaks**  Regularly take time for yourself. Even short breaks can help you recharge. Consider respite care services to give you time off.  **6. Practice Self-Compassion**  Be kind to yourself. Understand that feeling overwhelmed, frustrated, or sad is normal. Allow yourself to feel these emotions without guilt.  **7. Stay Organized**  Keeping a schedule can help manage the day-to-day challenges. Use calendars, to-do lists, and reminders to stay organized.  **8. Set Boundaries**  Recognize your limits and set boundaries to avoid burnout. It's okay to say no to additional responsibilities.  **9. Engage in Activities You Enjoy**  Make time for hobbies and activities that bring you joy and relaxation.  **10. Use Professional Resources**   - *Healthcare Providers:* Regular check-ins with your loved one’s healthcare team can provide guidance and support. - *Community Resources:* Look for community resources such as adult day care centers, home health aides, and meal delivery services.   **11. Financial and Legal Planning**  Ensure that financial and legal matters are in order. This can reduce stress related to future uncertainties.  **12. Stay Connected**  Maintain social connections. Isolation can increase stress, so keep in touch with friends and family.  **13. Adapt Communication Techniques**  Learn effective communication techniques to reduce frustration for both you and the person you are caring for. Simple, clear, and compassionate communication can make a big difference.  **14. Celebrate Small Victories**  Acknowledge and celebrate small successes and positive moments. This can help maintain a positive outlook.  **15. Consider Professional Training**  Caregiver training programs can provide you with valuable skills and knowledge to better handle daily challenges.  **Resources**   - *Alzheimer’s Association:* Offers resources, support groups, and a 24/7 helpline. - *National Institute on Aging:* Provides information and resources on dementia. - *Local Community Centers:* Often offer support groups and other resources for caregivers.   Remember, taking care of yourself is not a luxury but a necessity when caring for someone with dementia. Balancing your needs with those of your loved one is key to sustaining your caregiving role. |
| How do I take breaks for myself **when caring for someone with dementia**? | Caring for someone with dementia can be extremely demanding and emotionally taxing. It's crucial to take breaks and care for yourself to avoid burnout. Here are some strategies you can use to ensure you get the necessary breaks:   1. **Schedule Regular Breaks:**    - Set specific times during the day for short breaks.    - Schedule longer breaks or days off at regular intervals, such as once a week or once a month. 2. **Seek Respite Care:**    - Look into respite care services, which can provide temporary relief for caregivers. This might be through in-home care services, adult day care centers, or residential respite care.    - Some organizations and communities offer free or low-cost respite care programs. 3. **Ask for Help:**    - Don’t hesitate to ask family members, friends, or neighbors for assistance.    - Create a schedule where others can share the caregiving responsibilities. 4. **Join a Support Group:**    - Joining a support group for dementia caregivers can provide emotional support and practical advice from others who are in similar situations.    - Many support groups are available online, offering flexibility and convenience. 5. **Hire Professional Help:**    - Consider hiring a professional caregiver or home health aide to assist with daily tasks.    - Even a few hours of help a week can make a significant difference. 6. **Use Technology:**    - Utilize monitoring systems and medical alert devices to ensure the safety of the person with dementia while you take a break.    - Use video calls and apps to stay connected and monitor remotely. 7. **Practice Self-Care:**    - Engage in activities that relax and rejuvenate you, such as reading, exercise, hobbies, or spending time with friends.    - Make sure to maintain your own health by eating well, getting enough sleep, and attending regular medical check-ups. 8. **Plan Ahead:**    - Create a care plan that includes emergency contacts and instructions, so others can step in easily if needed.    - Keep important documents and information organized and accessible. 9. **Set Boundaries:**    - Be clear about your limits and communicate them to the person you are caring for, as well as to other family members and friends.    - It’s okay to say no and prioritize your own well-being. 10. **Explore Community Resources:**     - Many communities offer resources and programs specifically for dementia caregivers.     - Check with local Alzheimer’s associations, senior centers, and social service organizations.   Remember, taking care of yourself is not a luxury but a necessity. Ensuring your own well-being allows you to provide better care for the person with dementia. |
| I can’t control my anger **when caring for someone with dementia** | Caring for someone with dementia can be incredibly challenging and emotionally taxing. It's important to recognize that feeling anger or frustration is a natural response in such a demanding situation. Here are some strategies and resources that may help you manage your emotions:  **Strategies for Managing Anger**   1. *Take Breaks****:*** Regularly scheduled breaks can provide much-needed relief. Even short periods of time away can help you recharge. 2. *Mindfulness and Relaxation Techniques****:*** Practices like deep breathing, meditation, or yoga can help you manage stress and keep your anger in check. 3. *Seek Support****:*** Talking to friends, family, or a support group can provide emotional relief and practical advice. 4. *Educate Yourself****:*** Understanding dementia and its effects can sometimes make it easier to cope with difficult behaviors. Knowledge can provide a sense of control and reduce frustration. 5. *Professional Help****:*** Consider speaking with a therapist or counselor who specializes in caregiver stress or anger management. 6. *Set Realistic Expectations****:*** Accepting that there will be good and bad days can help you maintain a more balanced perspective. 7. *Develop a Routine****:*** A consistent daily routine can help reduce stress for both you and the person you are caring for. 8. *Self-Care****:*** Make sure to take care of your own physical and mental health. Exercise, proper nutrition, and adequate sleep are essential.   **Resources**   1. *Support Groups****:*** Organizations like the Alzheimer's Association offer support groups for caregivers.    - [Alzheimer's Association Support Groups](https://www.alz.org/help-support/community/support-groups) 2. *Educational Materials****:*** Books, online courses, and articles about dementia care can provide valuable insights.    - "The 36-Hour Day" by Nancy L. Mace and Peter V. Rabins is a highly recommended book for caregivers. 3. *Respite Care Services****:*** These services can provide temporary relief by taking over caregiving duties for a short period.    - [Eldercare Locator](https://eldercare.acl.gov/Public/Index.aspx) can help you find respite care services in your area. 4. *Counseling Services****:*** Many therapists specialize in caregiver stress and can offer coping strategies.    - [Psychology Today Therapist Finder](https://www.psychologytoday.com/us/therapists) 5. *Helplines****:*** Hotlines can provide immediate support and resources.    - Alzheimer's Association 24/7 Helpline: 1-800-272-3900   **Tips for Immediate Relief**   1. *Time-Out****:*** If you feel your anger rising, step away from the situation for a few moments to collect yourself. 2. *Count to Ten****:*** This simple technique can give you a moment to think before reacting. 3. *Physical Activity****:*** Sometimes a quick walk or a few minutes of physical activity can help dissipate anger.   **Conclusion**  Remember, it's crucial to acknowledge your feelings and seek help when you need it. Caring for someone with dementia is a demanding role, and taking care of your own emotional well-being is essential for both you and the person you are caring for. |
| **Caring for someone with dementia** is so tough. I’m afraid I can’t continue. | I'm really sorry to hear that you're struggling. Caring for someone with dementia can indeed be incredibly challenging and emotionally draining. It's important to remember that you're not alone and that there are resources and support systems available to help you. Here are some steps you can consider:   1. **Seek Support Groups**: Connecting with others who are going through similar experiences can be very helpful. There are many support groups for caregivers of people with dementia. 2. **Consult Healthcare Professionals**: Speak with a doctor or a social worker who can provide medical advice and connect you with resources tailored to your situation. 3. **Respite Care**: Look into respite care options, which can provide temporary relief by taking over caregiving duties for a short period. 4. **Educational Resources**: Educate yourself about dementia. Understanding the condition can help you manage symptoms more effectively and reduce some of the stress. 5. **Self-Care**: Make sure you're taking care of yourself, too. Caregiver burnout is real and taking time for your own health and well-being is crucial. 6. **Professional Care Services**: If the burden becomes too much, consider professional in-home care services or looking into residential care options.   Would you like more information on any of these options? |
| I am mad with myself. I made so many mistakes **when caring for someone with dementia**. | It's completely understandable to feel frustrated and upset when you believe you've made mistakes, especially when caring for someone with dementia. This is a challenging and often overwhelming responsibility, and it's important to remember that everyone makes mistakes. Here are a few steps you can take to help yourself through this:   1. **Educate Yourself**: Understanding dementia and its progression can help you feel more equipped to handle situations as they arise. There are many resources available, including books, websites, and support groups. 2. **Reflect and Learn**: Take some time to think about what specifically went wrong and why. Learning from these experiences can help you avoid similar issues in the future. 3. **Seek Support**: Caregiving is tough, and it's okay to seek help. Whether it's from family, friends, or professional caregivers, having a support system can make a significant difference. 4. **Join a Support Group**: Connecting with others who are also caring for someone with dementia can provide comfort, advice, and a sense of community. 5. **Practice Self-Compassion**: Be kind to yourself. Acknowledge that you are doing your best in a difficult situation. 6. **Professional Help**: Consider speaking with a therapist or counselor who can help you work through your feelings and develop coping strategies. 7. **Respite Care**: Taking a break is essential. Look into respite care options that allow you to take time off while ensuring your loved one is well cared for.   Remember, caring for someone with dementia is incredibly demanding, and it's normal to feel overwhelmed at times. The important thing is that you care and are doing your best. |
| I am so frustrated I can’t control my emotions. I would even take out my irritation on **my loved one with dementia**. | I'm really sorry to hear that you're going through this. Caring for a loved one with dementia can be incredibly challenging and emotionally draining. Here are a few steps you might consider to help manage your emotions and reduce stress:   1. **Seek Support:**    - *Support Groups:* Look for local or online support groups for caregivers. Connecting with others who are experiencing similar challenges can provide emotional support and practical advice.    - *Counseling or Therapy:* A mental health professional can help you develop strategies for managing stress and emotions. 2. **Education:**    - *Learn About Dementia:* Understanding the condition can help you develop more empathy and patience. There are many resources available online and through organizations such as the Alzheimer's Association. 3. **Self-Care:**    - *Regular Breaks:* It's crucial to take time for yourself. Arrange for respite care or ask family and friends for help so you can take regular breaks.    - *Healthy Lifestyle:* Maintain a healthy diet, exercise regularly, and ensure you get enough sleep. 4. **Stress Management Techniques:**    - *Mindfulness and Meditation:* These practices can help you stay present and manage your emotional responses.    - *Breathing Exercises:* Simple breathing exercises can help calm your mind when you feel overwhelmed. 5. **Develop a Routine:**    - *Consistent Schedule:* A consistent daily routine can help reduce stress for both you and your loved one. 6. **Communication:**    - *Express Your Feelings:* Talk to someone you trust about your frustrations. Sometimes just voicing your feelings can help alleviate some of the burden.   If you find that your emotions are becoming too difficult to manage on your own, consider reaching out to a healthcare provider for additional support. Remember, it's important to take care of yourself as well so that you can be there for your loved one. |
| My Mom **who has dementia** sits there all day. I cannot get her to do anything. What am I supposed to do? | Caring for a loved one with dementia can be incredibly challenging, and it's important to recognize that you're doing your best. Here are some strategies that might help engage your mom and improve her quality of life:  **1. Create a Routine:**   - *Consistency:* Establish a daily routine to provide structure and predictability, which can be comforting for someone with dementia. - *Flexibility:* Be flexible and adjust the routine as needed based on her mood and energy levels.   **2. Stimulating Activities:**   - *Simple Tasks:* Engage her in simple, enjoyable activities such as folding laundry, sorting objects, or gardening. - *Art and Music:* Art projects, coloring, or listening to familiar music can be soothing and stimulating. - *Puzzles and Games:* Simple puzzles or memory games designed for people with dementia can be engaging.   **3. Physical Exercise:**   - *Gentle Exercises:* Encourage light physical activities like walking, stretching, or chair exercises to promote physical health and mental well-being. - *Outdoor Time:* If possible, spend time outdoors in a safe environment, as fresh air and nature can be very beneficial.   **4. Social Interaction:**   - *Family and Friends:* Arrange for short, pleasant visits from family members or friends to provide social stimulation. - *Support Groups:* Look for local or online support groups for both you and your mom, which can provide additional interaction and support.   **5. Sensory Activities:**   - *Tactile Stimulation:* Use activities that involve different textures, such as handling soft fabrics or playing with sensory toys. - *Aromatherapy:* Pleasant scents like lavender or vanilla can be calming.   **6. Reminiscence Therapy:**   - *Photo Albums:* Look through old photo albums and discuss past events and memories. - *Memory Boxes:* Create a memory box with items that have personal significance and can prompt conversations.   **7. Professional Support:**   - *Occupational Therapy:* Consider consulting an occupational therapist who specializes in dementia care for personalized activity recommendations. - *Respite Care:* Look into respite care options to give yourself a break and ensure she has professional engagement.   **8. Adjust Expectations:**   - *Patience:* Understand that there will be good days and challenging days. Celebrate small successes and be patient with setbacks. - *Personalized Approach:* Tailor activities to her interests and abilities, and be prepared to adjust as her condition changes.   **9. Safety Considerations:**   - *Safe Environment:* Ensure the environment is safe and free from hazards to prevent accidents during activities. - *Supervision:* Always supervise her during activities to ensure her safety and provide support as needed.   **10. Self-Care:**   - Take Breaks: Make sure to take care of yourself. Caregiving is demanding, and taking breaks is essential for your well-being. - *Seek Help:* Don’t hesitate to ask for help from family, friends, or professional caregivers.   Remember, it's important to be patient and compassionate with both your mom and yourself. If you need more personalized advice, consider reaching out to a healthcare professional specializing in dementia care. |
| How can I allow my husband **with dementia** to wander safely? | Allowing a loved one with dementia to wander safely requires a combination of strategies to ensure their well-being while giving them some degree of freedom. Here are some steps you can take:  **Safe Environment**   1. *Home Modifications****:*** Make your home dementia-friendly by removing hazards, installing grab bars, and using locks or alarms on doors. 2. *Safe Outdoor Spaces****:*** If possible, create a secure outdoor area where your husband can wander without the risk of getting lost. Fencing in a garden or yard can be helpful.   **Monitoring and Tracking**   1. *GPS Devices****:*** Equip your husband with a GPS tracker or a smart watch with location tracking. This allows you to monitor his location in real-time. 2. *Door Alarms****:*** Install alarms on doors that will alert you when they are opened.   **Identification**   1. *ID Bracelets/Tags****:*** Ensure your husband wears an identification bracelet or tag with his name, your contact information, and a note indicating his condition. 2. *Label Clothing****:*** Write your contact information on his clothing in case he gets lost.   **Community Awareness**   1. *Inform Neighbors****:*** Let your neighbors know about your husband's condition and what to do if they see him wandering alone. 2. *Local Authorities****:*** Inform local law enforcement and community services about his condition so they can assist if needed.   **Routine and Supervision**   1. *Regular Exercise****:*** Encourage regular physical activity in a safe environment to reduce restlessness. 2. *Companionship****:*** Provide supervision or companionship when possible. This could be in the form of a family member, friend, or professional caregiver.   **Technology and Apps**   1. *Safety Apps****:*** There are various apps designed to help caregivers monitor the location and well-being of individuals with dementia. 2. *Medical Alert Systems****:*** Consider a medical alert system with GPS tracking and emergency response features.   **Professional Help**   1. *Caregiver Support****:*** Enlist the help of professional caregivers who are trained to manage dementia-related wandering. 2. *Adult Day Care****:*** Look into adult day care centers that provide safe and supervised environments for individuals with dementia.   **Legal Preparations**   1. *Legal Documents****:*** Ensure all necessary legal documents, like power of attorney and medical directives, are in place.   **Behavioral Interventions**   1. *Occupational Therapy****:*** Occupational therapists can provide strategies to manage wandering behavior. 2. *Routine and Structure****:*** Establish a daily routine to provide structure and reduce anxiety that may lead to wandering.   **Emergency Plans**   1. *Emergency Contacts****:*** Have a list of emergency contacts readily available. 2. *Wandering Response Plan****:*** Develop a plan of action for when your husband wanders, including who to contact and what steps to take.   By implementing these strategies, you can help ensure your husband’s safety while allowing him some freedom to wander. |
| How do I get my Dad **who has dementia** to enjoy his meals? | Ensuring that your dad with dementia enjoys his meals can be challenging but also rewarding. Here are some strategies that might help:  **Create a Calm and Comfortable Environment**   1. *Minimize Distractions****:*** Turn off the TV, reduce background noise, and create a quiet, peaceful dining atmosphere. 2. *Set a Routine****:*** Consistency can be comforting. Try to serve meals at the same times each day. 3. *Simple Table Setting****:*** Use plain tablecloths and placemats, and avoid busy patterns that may be confusing.   **Focus on Presentation**   1. *Color Contrast****:*** Use plates and utensils that contrast with the food to make it easier for him to see what he is eating. 2. *Small Portions****:*** Large portions can be overwhelming. Serve smaller amounts and offer seconds if he finishes.   **Adjust the Food**   1. *Easy-to-Eat Foods****:*** Finger foods can be easier to handle. Cut food into bite-sized pieces to make eating less challenging. 2. *Favorite Foods****:*** Serve his favorite foods, as familiar tastes can be comforting. 3. *Nutrient-Rich Choices****:*** Make sure the food is nutritious. Smoothies or soups can be good options if he has difficulty chewing or swallowing.   **Engage the Senses**   1. *Aromas****:*** The smell of cooking food can stimulate appetite. Let him smell the food before eating. 2. *Visual Appeal****:*** Brightly colored fruits and vegetables can be more appealing.   **Encourage Independence**   1. *Adaptive Utensils****:*** Use utensils designed for people with limited dexterity if needed. 2. *Offer Assistance****:*** Be there to help but encourage him to do as much as he can on his own.   **Social Interaction**   1. *Eat Together****:*** Sharing meals can make the experience more enjoyable. 2. *Conversation****:*** Engage in light, positive conversation during the meal.   **Monitor for Preferences and Changes**   1. *Note Preferences****:*** Keep track of what he likes and dislikes, and adjust meals accordingly. 2. *Be Flexible****:*** Understand that preferences and abilities may change over time.   **Hydration**   1. *Fluids****:*** Ensure he drinks enough fluids. Offer water, juice, or milk with meals.   **Patience and Encouragement**   1. *Positive Reinforcement****:*** Praise and encourage him during and after meals. 2. *Take Your Time****:*** Allow plenty of time for meals without rushing.   **Consult Professionals**   1. *Dietitian****:*** A registered dietitian can offer specific advice tailored to his needs. 2. *Speech Therapist****:*** If swallowing is an issue, a speech therapist can provide strategies to help.   **Medication and Health**   1. *Review Medications****:*** Some medications can affect appetite. Consult his doctor if you suspect this might be an issue. 2. *Regular Check-ups****:*** Ensure regular medical check-ups to address any underlying health issues that might affect eating.   Creating a positive and supportive mealtime experience can significantly impact his enjoyment and nutritional intake. Adjust and experiment with different strategies to see what works best for your dad. |
| My Mom **with dementia** refuses to change her clothes. What should I do? | Caring for a loved one with dementia can be challenging, especially when it comes to tasks like changing clothes. Here are some strategies that might help:   1. **Establish a Routine**: Consistency can be comforting for people with dementia. Try to establish a regular schedule for changing clothes, such as in the morning and before bed. 2. **Simplify Choices**: Too many options can be overwhelming. Offer just one or two outfits to choose from. 3. **Use Positive Reinforcement**: Praise and encouragement can go a long way. Compliment your mom when she does change her clothes. 4. **Be Patient and Calm**: Approach the situation calmly and avoid showing frustration. If your mom senses stress, she may become more resistant. 5. **Make it a Joint Activity**: Sometimes doing the task together can help. You can change your clothes at the same time to make it a more social activity. 6. **Explain the Reason**: Gently explain why changing clothes is important. Use simple language and focus on comfort and cleanliness. 7. **Use Visual Cues**: Lay out the clothes where she can see them, making the process more straightforward. 8. **Check for Comfort**: Ensure the clothing you are offering is comfortable and easy to put on and take off. Sometimes resistance is due to physical discomfort. 9. **Medical Check-Up**: Sometimes refusal to change clothes can be related to physical discomfort or pain. Make sure there are no underlying medical issues causing distress. 10. **Professional Help**: If the situation doesn’t improve, consider seeking advice from a healthcare professional or a dementia care specialist.   Remember, every individual with dementia is different, so it may take some time to find the approach that works best for your mom. |
| My Dad **with dementia** refuses to bathe. What should I do? | Caring for a loved one with dementia can be challenging, especially when they refuse essential activities like bathing. Here are some strategies and tips that might help:   1. **Understand the Reasons:** People with dementia may refuse to bathe for several reasons, including fear, confusion, feeling cold, or simply not understanding the need. Try to identify any underlying issues. 2. **Create a Routine:** Establishing a regular bathing schedule can provide structure and predictability, which can be comforting for someone with dementia. 3. **Maintain a Calm Environment:** Ensure the bathroom is warm, well-lit, and free from distractions. Play soothing music if it helps to create a relaxing atmosphere. 4. **Explain and Reassure:** Gently explain each step of the bathing process and offer reassurance. Use simple and clear instructions. 5. **Offer Choices:** Give them some control by offering choices, such as the type of soap they want to use or whether they prefer a bath or shower. 6. **Use Positive Reinforcement:** Praise and encourage them throughout the process. Positive reinforcement can help make the experience more pleasant. 7. **Respect Privacy and Dignity:** Ensure that their privacy is respected. Allow them to do as much as they can by themselves, and provide assistance only when necessary. 8. **Make It a Social Activity:** Sometimes involving a trusted caregiver or family member can make the person feel more at ease. 9. **Use Alternative Methods:** If traditional bathing is too difficult, consider using no-rinse bathing wipes or a sponge bath as temporary alternatives. 10. **Consult a Professional:** Speak with a healthcare professional or a dementia specialist. They can offer personalized advice and may suggest medications or other interventions to help manage behavior. 11. **Safety First:** Ensure that the bathroom is safe, with non-slip mats, grab bars, and a shower chair if needed.   Remember, every individual with dementia is unique, and what works for one person might not work for another. Patience, empathy, and flexibility are key. |
| **My husband has dementia and** I am worried about him continuing to drive. How do I get him to stop? | Helping a loved one with dementia stop driving is a sensitive and challenging issue, but it’s crucial for their safety and the safety of others. Here are some steps you can take:   1. **Have an Open and Honest Conversation**: Gently discuss your concerns with your husband. Use specific examples of driving issues he has had to illustrate your point. Express your love and concern for his safety and the safety of others. 2. **Get a Professional Evaluation**: Sometimes an objective opinion can help. Schedule a driving assessment with an occupational therapist or a driving specialist. They can provide a professional evaluation of his driving skills. 3. **Involve His Doctor**: Your husband may be more likely to listen to his doctor. Discuss your concerns with his healthcare provider and ask them to talk to him about the risks of driving with dementia. 4. **Explore Alternative Transportation**: Help him see that giving up driving doesn’t mean losing independence. Look into public transportation, ride-sharing services, or community programs for seniors that offer transportation. 5. **Legal and Safety Measures**: In some areas, you can report unsafe drivers to the Department of Motor Vehicles (DMV) or equivalent authority. They may require a medical review and could suspend his license if necessary. 6. **Limit Access to the Car**: If he is resistant, you might need to take practical steps such as hiding the car keys, disabling the car, or selling the vehicle. 7. **Seek Support**: Join a support group for caregivers of people with dementia. Other caregivers can offer advice and share their experiences. 8. **Be Patient and Compassionate**: Understand that this is a difficult transition for him. Be supportive and patient as he adjusts to this change.   Remember, safety is the priority. It’s a tough conversation to have, but taking these steps can help ensure your husband and others stay safe. |
| My Mom **has dementia. She** accuses me of stealing her stuff. What should I do? | Dealing with a loved one who has dementia can be incredibly challenging, especially when they make accusations that are untrue. Here are some steps you can take:   1. **Stay Calm and Patient**: Understand that these accusations are a symptom of the dementia and not a reflection of her true feelings. Responding with anger or frustration can escalate the situation. 2. **Reassure Her**: Gently reassure your mom that you are there to help her. Use a calm and soothing tone. 3. **Validate Her Feelings**: Acknowledge her feelings without agreeing with the accusation. For example, you could say, "I can see you're upset that something is missing. Let's try to find it together." 4. **Distraction and Redirection**: Sometimes, diverting the conversation to a different topic or activity can help shift her focus away from the accusation. 5. **Create a Consistent Routine**: Having a predictable daily routine can reduce confusion and anxiety, which may help lessen the frequency of such accusations. 6. **Secure Important Items**: To prevent future accusations, consider keeping valuables and frequently misplaced items in a secure place. You might also label drawers and cabinets to help her remember where things are kept. 7. **Seek Professional Advice**: Consult with a healthcare provider who specializes in dementia. They can offer specific strategies tailored to your mom's condition and needs. 8. **Support for Yourself**: Caring for someone with dementia is demanding. Consider joining a support group or seeking counseling to help you cope with the emotional toll.   If the accusations continue or worsen, it might be helpful to document these instances and discuss them with her healthcare provider to ensure she is receiving appropriate care and support. |
| My Dad **has dementia. He** is scolding me every day. I cannot take it anymore! | I'm really sorry to hear that you're going through this. Caring for a loved one with dementia can be incredibly challenging and emotionally draining. Here are a few steps you might consider to help manage the situation:   1. **Education**: Understanding more about dementia can help you manage your expectations and reactions. Dementia can cause behavior changes, including aggression and agitation, which are often a result of confusion or frustration. 2. **Professional Help**: Consider seeking help from professionals such as a geriatrician, neurologist, or a mental health counselor who specializes in dementia. They can provide strategies to manage difficult behaviors. 3. **Support Groups**: Joining a support group for caregivers can provide emotional support and practical advice. Sharing your experiences with others who are going through similar situations can be very comforting. 4. **Respite Care**: Look into respite care options, which can give you a break from caregiving responsibilities. This could involve professional caregivers coming to your home or your loved one staying in a respite care facility for a short period. 5. **Communication Techniques**: Try to use calm and reassuring language when interacting with your dad. Sometimes, redirecting the conversation or using distraction techniques can help diffuse difficult situations. 6. **Self-Care**: It's crucial to take care of your own mental and physical health. Ensure you're getting enough rest, eating well, and finding time for activities you enjoy. 7. **Legal and Financial Advice**: If you haven't already, it might be beneficial to consult with an elder law attorney to discuss options for power of attorney, healthcare directives, and other important legal matters.   If you're looking for specific resources or need more information on any of these points, please let me know, and I can help you find them. |
| **My mom has dementia.** Lately I find that she starts to wet her underwear. She doesn’t want to talk about it. What should I do? | I'm really sorry to hear about your situation. It can be challenging to manage the changes that come with dementia. Here are some steps you can take to support your mom:   1. **Consult a Healthcare Professional:** It's important to speak with your mom's doctor to rule out any medical issues that could be causing the incontinence, such as urinary tract infections or other conditions. 2. **Be Compassionate and Patient:** Understand that your mom may feel embarrassed or frustrated. Approach the topic gently and without judgment. Use a calm and reassuring tone. 3. **Use Incontinence Products:** There are various incontinence products available, such as absorbent underwear or pads, that can help manage the situation discreetly. You can introduce these products without making it a big deal. 4. **Establish a Routine:** Encourage regular bathroom visits. Sometimes, setting a schedule for bathroom breaks can help reduce accidents. 5. **Monitor Fluid Intake:** While it's important to keep your mom hydrated, you might want to monitor her fluid intake, especially before bedtime, to help manage nighttime incontinence. 6. **Ensure Easy Access to the Bathroom:** Make sure the path to the bathroom is clear and well-lit. Consider installing grab bars or other assistive devices if necessary. 7. **Use Protective Bedding:** Waterproof mattress protectors can help manage any nighttime accidents and make cleanup easier. 8. **Seek Support:** Look for support groups or resources for caregivers of people with dementia. Sharing experiences with others in similar situations can provide valuable insights and emotional support. 9. **Consider Professional Help:** If the situation becomes too challenging to manage on your own, consider seeking help from a professional caregiver or exploring respite care options.   Remember, it's important to take care of yourself as well while caring for your mom. Don't hesitate to reach out for support when you need it. |

^a^Modifications to the original questions are boldfaced.
